# Effect of B7-H4 downregulation induced by *Toxoplasma gondii* infection on dysfunction of decidual macrophages contributes to adverse pregnancy outcomes

**DOI:** 10.1186/s13071-022-05560-9

**Published:** 2022-12-13

**Authors:** Lijun Cui, Yu Wang, Liqin Ren, Zhidan Li, Yuzhu Jiang, Chao Wang, Xianbing Liu, Yushan Ren, Xuemei Hu

**Affiliations:** 1 Department of Immunology, Binzhou Medical University, Yantai, 264003 Shandong People’s Republic of China; 2 Department of Medical Genetics and Cell Biology, Binzhou Medical University, Yantai, 264003 Shandong People’s Republic of China

**Keywords:** *Toxoplasma gondii*, Decidual macrophage, B7-H4, Abnormal pregnancy, Adoptive transfer

## Abstract

**Background:**

*Toxoplasma gondii *infection during pregnancy can lead to fetal defect(s) or congenital complications. The inhibitory molecule B7-H4 expressed on decidual macrophages (dMφ) plays an important role in maternal–fetal tolerance. However, the effect of B7-H4 on the function of dMφ during *T. gondii* infection remains unclear.

**Methods:**

Changes in B7-H4 expression on dMφ after *T. gondii* infection were explored both in vivo and in vitro. B7-H4^-/-^ pregnant mice (pregnant mice with B7-H4 gene knockout) and purified primary human dMφ treated with B7-H4 neutralizing antibody were used to explore the role of B7-H4 signaling on regulating the membrane molecules, synthesis of arginine metabolic enzymes and cytokine production by dMφ with *T. gondii* infection. Also, adoptive transfer of dMφ from wild-type (WT) pregnant mice or B7-H4^-/-^ pregnant mice to infected B7-H4^-/-^ pregnant mice was used to examine the effect of B7-H4 on adverse pregnancy outcomes induced by *T. gondii* infection.

**Results:**

The results illustrated that B7-H4^-/-^ pregnant mice infected by *T. gondii* had poorer pregnancy outcomes than their wild-type counterparts. The expression of B7-H4 on dMφ significantly decreased after *T. gondii* infection, which resulted in the polarization of dMφ from the M2 toward the M1 phenotype by changing the expression of membrane molecules (CD80, CD86, CD163, CD206), synthesis of arginine metabolic enzymes (Arg-1, iNOS) and production of cytokines (IL-10, TNF-α) production. Also, we found that the B7-H4 downregulation after *T. gondii* infection increased iNOS and TNF-α expression mediated through the JAK2/STAT1 signaling pathway. In addition, adoptive transfer of dMφ from a WT pregnant mouse donor rather than from a B7-H4^-/-^ pregnant mouse donor was able to improve adverse pregnancy outcomes induced by *T. gondii* infection.

**Conclusions:**

The results demonstrated that the downregulation of B7-H4 induced by *T. gondii* infection led to the dysfunction of decidual macrophages and contributed to abnormal pregnancy outcomes. Moreover, adoptive transfer of B7-H4^+^ dMφ could improve adverse pregnancy outcomes induced by *T. gondii* infection.

**Graphical Abstract:**

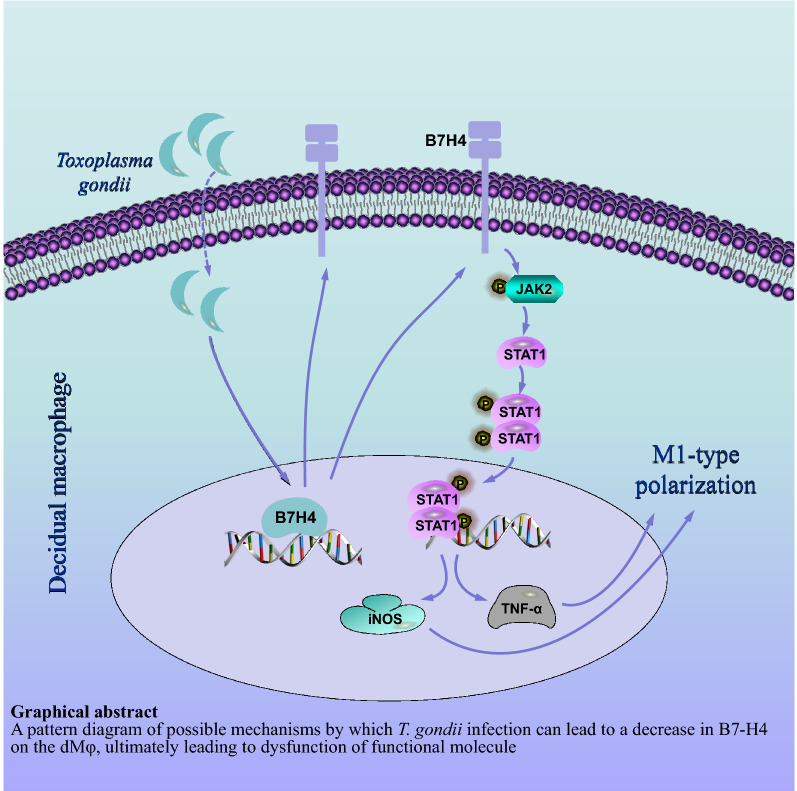

**Supplementary Information:**

The online version contains supplementary material available at 10.1186/s13071-022-05560-9.

## Background

*Toxoplasma gondii* is an obligate intracellular protozoan parasite that infects nearly one-third of the world’s human population [1]. Although most infections are typically asymptomatic, *T. gondii* can cause severe zoonotic toxoplasmosis in immunocompromised individuals, such as pregnant women [2, 3]. Women in early pregnancy, in particular, once infected, can experience miscarriage, premature delivery, stillbirth and other adverse pregnancy outcomes [4]. Previous studies suggested that the abnormal pregnancy induced by *T. gondii* infection is closely connected with the disruption of the immune microenvironment at the maternal–fetal interface [5, 6]. The immune microenvironment comprises various decidual immune cells (natural killer cells, macrophages, dendritic cells and regulatory T cells) and their immune-regulatory molecules, all of which establish specific maternal immune tolerance to the semi-allogeneic fetus during normal pregnancy [7].

Decidual macrophages (dMφ) are the second-most abundant immune cell at the maternal–fetal interface, accounting for 20–25% of decidual immune cells, and participate in implantation, trophoblast invasion and fetal development during pregnancy [8]. Early studies performed by our group have substantiated that the dysfunction of dMφ induced by *T. gondii* infection contributes to adverse pregnancy outcomes [9]. We also found that some inhibitory receptors, such as LILRB4 and Tim-3, could skew macrophage polarization towards the classically activated M1 phenotype and lead to serious adverse pregnancy outcomes during *T. gondii* infection [10, 11]. Results from recent studies indicate that another novel inhibitory molecule, B7-H4, which is mainly expressed on macrophages and dendritic cells, benefit maternal–fetal tolerance during normal pregnancy [12–14]. Our most recent study confirmed that B7-H4 is downregulated on decidual dendritic cells after *T. gondii* infection, eventually leading to the dysfunction of the decidual dendritic cells [15]. However, we were unable to determine whether *T. gondii* infection can affect the expression of B7-H4 and result in the dMφ function disorder.

B7-H4, a member of the B7 family, was discovered in 2003 and found to inhibit the activation of immune cells [16]. B7-H4 has been shown to be expressed by Mφ and to possibly favor the tolerant phenotype and contribute to a normal pregnancy [17]. Subsequent studies showed that B7-H4 expression is higher in M2 cells than in M1 cells [18, 19] and that the main function of M2 cells is immunosuppressive, promoting immune tolerance at the maternal–fetal interface [20]. Our early research indicated that *T. gondii* infection was responsible for the switch from the M2 phenotype of dMφ toward the M1 phenotype, which resulted in disruption of the immunosuppressive microenvironment at the maternal–fetal interface and contributed to adverse pregnancy outcomes [9]. In addition, following *T. gondii* infection, the expression of CD206, CD209, CD163, arginase-1 (Arg-1) and interleukin-10 (IL-10), all considered to be markers of the M2 phenotype, by dMφ decreased, whereas the expression of CD80, CD86, inducible nitric oxide synthase (iNOS) and tumor necrosis factor alpha (TNF-α), all M1 markers, increased [10, 11]. However, whether *T. gondii* infection affects the expression of B7-H4 on dMφ and results in the latter’s dysfunction has not been reported. In the present study, the change in B7-H4 expression on dMφ after *T. gondii* infection was explored. We also investigated how B7-H4 signaling regulates the membrane molecules, synthesis of arginine metabolic enzymes, and dMφ expression of cytokines with *T. gondii* infection.

It has been shown that B7-H4 is able to negatively modulate the phosphorylation of signal transducer and activator of transcription 1 (STAT1) in monocytes infected with human cytomegalovirus [21]. Moreover, the results of many studies suggest that the Janus kinase 2 (JAK2)/STAT1 signaling pathway is essential for polarization of the M1 phenotype, as evidenced by increased iNOS and TNF-α production [22, 23]. iNOS is an important marker of the M1-type macrophage and is upregulated when the M1-type macrophage is activated. iNOS has been found to catalyze the reaction between l-arginine and oxygen molecules to produce a large amount of nitric oxide (NO), while a high concentration of NO was pro-inflammatory [24]. Excessive production of iNOS may suppress placental vascular development and give rise to early embryo loss [25]. TNF-α, as an important pro-inflammatory cytokine, is mainly secreted by macrophages; it could activate neutrophils and lymphocytes and plays a key role in the upstream initiation phase of the inflammatory cascade [26]. In pregnancy, a normal physiological concentration of TNF-α is beneficial to the pregnancy, but excessive secretion of TNF-α will cause the maternal–fetal interface immune disorder, such as T helper cell 1/T helper cell 2 (Th1/Th2) imbalance, macrophage changes and enhanced natural killer (NK) cell killing ability, eventually resulting in abortion, premature delivery and other adverse pregnancy outcomes [27]. However, whether the change in B7-H4 expression following *T. gondii* infection could modulate the expression of iNOS and TNF-α in dMφ via the JAK2/STAT1 signaling pathways also remains unclear.

In the present study, primary human dMφ and B7-H4^-/-^ pregnant mice (pregnant mice with B7-H4 gene knockout) were used to investigate the role of B7-H4 in regulating dMφ functions in adverse pregnancy outcomes caused by *T. gondii.* We also examined the adoptive transfer of dMφ collected from wild-type (WT) pregnant mice or B7-H4^-/-^ pregnant mice to infected B7-H4^-/-^ pregnant mice to examine the effect of B7-H4 on adverse pregnancy outcomes induced by *T. gondii* infection.

## Methods

### Animals

C57/BL6 mice (Jinan Pengyue Laboratory Animal Breeding Co. Ltd., Jinan, China) and B7-H4^-/-^ mice (Nanjing Institute of Biomedicine, Nanjing, China) were bred and maintained with sufficient sterilized food and water, under conditions of controlled temperature (20–24 °C) and humidity (40%–60%) and a 12/12-h light/dark cycle in the specific-pathogen-free animal unit. Females (age 6–8 weeks) were mated to males (age 8–10 weeks) at a ratio of 2:1 overnight. The next morning, those females with vaginal plugs (gestational day [Gd] 0) were segregated. WT C57/BL6 pregnant mice were randomly divided into an uninfected and infected group, and B7-H4^-/-^ infected mice were used as the infected B7-H4^-/-^ group. Each group consisted of 10 mice. On Gd 8, pregnant mice in the WT infected group and B7-H4^-/-^ infected group were intraperitoneally injected with 400 tachyzoites of *T. gondii* strain RH in 200 μl sterile phosphate-buffered saline (PBS). The uninfected mice were intraperitoneally injected with 200 μl sterile PBS at the same time. For supplementary details, see Additional file 1: Text S1.

### Preparation of* T. gondii* (RH strain)

The tachyzoites (RH strain) stored in liquid nitrogen were retrieved, and the frozen storage tube was shaken rapidly in a water bath box at 40 °C. After complete dissolution, the frozen solution was transferred to a tube with a threefold higher volume of sterile PBS, washed twice and cultured in HEp-2 cell lines with Minimum Essential Medium (BC-—020; Bio Channel, China) supplemented with 5% fetal bovine serum (FBS; #A3160801; Gibco, Thermo Fisher Scientific,Waltham, MA, USA) and 100 IU/ml penicillin/streptomycin (#P1400; Solarbio Science & Technology Co., Ltd., Beijing, China). After 54 h of culture, HEp-2 cells were centrifuged at 400 × *g* for 10 min 4 °C, and the clear supernatant was then centrifuged at 2800 × *g* for 7 min at 4 °C to collect the tachyzoites. The purified tachyzoites were counted in a Neubauer chamber and cultured with new HEp-2 cells.

### Acquisition and identification of homozygous B7-H4^***-/-***^*** mice***

The B7-H4^-/-^ mice were successfully bred by the Nanjing University-Nanjing Institute of Biomedicine with the background of C57BL/6. At the age of 4 weeks, the tail of each mouse was cut, and the DNA of the tail tissue was obtained using a DNA extraction kit (#2003G24; Generay, Shanghai, China). The DNA was used as a template for real-time PCR amplification, following which the products were sent to the Shanghai Meiji Biomedical Technology Co. Ltd. (Shanghai, China) for DNA sequencing to obtain homozygous mice with the B7-H4 gene knockout. The homozygous B7-H4^-/-^ mice were continuously cultivated to guarantee the establishment of an animal model with adverse pregnancy outcomes in infected B7-H4^-/-^ mice.

### Scanning electron microscopy

Mice were sacrificed on Gd 14 and dissected. All fetuses were carefully removed, washed 5–6 times with 0.1 M phosphate buffer and then fixed with 2.5% phosphate buffer glutaraldehyde for 48 h at 4 °C. The immobilized fetus was dehydrated using a graded ethanol series and soaked for 10 min at a time. The sample was dried in the K850 Critical Point Dryer (Quorum Technologies Ltd., Lewes, UK), attached to the sample support, and gold-coated with Quorum Q150RS coating system (Quorum Technologies Ltd.). All samples were observed with a 10 kV scanning electron microscope (EVO LS15l; Carl Zeiss A.G., Oberkochen, Germany). Images were obtained using the SmartSEM user interface software (Carl Zeiss A.G.).

### Hematoxylin–eosin staining

Pregnant mice were sacrificed on Gd 14 and dissected. All fetuses were carefully removed and each placenta exposed to 4% paraformaldehyde for 1 week, following which they were placed in a specimen box and rinsed with running water for 4–12 h and then dehydrated in a dehydrator. After paraffin embedding, the placenta was cut into 5-µm-thick slices and baked at 55–60 °C for 3–10 h. The sample was then subjected to xylene dewaxing for 5 times, with each dewaxing lasting for 5–10 min. Following dewaxing, the placenta was dehydrated in an ethanol gradient and then soaked 3 times in steaming water for 3 min each time. Harris hematoxylin staining was conducted for 10 min, and the slices were then rinsed 3 times in steaming water for several seconds each time. Next, 0.5% hydrochloric acid alcohol separation was conducted for 3–10 s, and eosin staining was performed for 2 min. After fixing with xylene, the slides were sealed with neutral resin, covered with a cover glass and observed and photographed under a microscope.

### Single-cell preparation of mouse

Pregnant uninfected, infected and B7-H4^-/-^ infected mice on Gd 14 were sacrificed by cervical dislocation. Mouse uteri and placentas were carefully separated and dissected with scissors to remove fetuses and then rinsed twice with sterile cold PBS. The placentas and uteri were cut into small pieces and shredded carefully by using the gentleMACS Dissociator (Miltenyi Biotec, Bergisch Gladbach, Germany). The tissue suspension was filtered through a 75-µm sterile screen, and the single-cell suspension was obtained by gently grinding the needle bolt of the glass syringe as an abrasive rod. The single-cell suspension was centrifuged with Ficoll density gradient at 400 × *g* for 20 min, and the white film layer was centrifuged at 400 × *g* for 10 min to remove the impurities. The mouse decidual mononuclear cells were resuspended in 100–200 μl PBS and then analyzed by flow cytometry. The mouse carcasses were stored in a freezer at − 20 °C and disposed by professional organizations.

### Adoptive transfer experiment

The B7-H4^-/-^ and C57/BL6 pregnant mice were sacrificed serially by cervical dislocation on Gd 12. Single-cell suspensions were separately prepared from the placental and uterine tissues by cutting the tissues into small pieces and filtering them through a 75-µm sterile nylon mesh. The mononuclear cells were isolated by Ficoll density gradient centrifugation. F4/80^+^ macrophages were positively selected using the Mouse F4/80 Positive Selection Kit (#8802–6863; Thermo Fisher Scientific) according to the manufacturer’s instructions. The purified macrophages were centrifuged at 400 × *g* for 10 min and then labeled with 15 µM carboxyfluorescein succinimidyl amino ester (CFSE) (#HY-D0938; MedChemExpress, Monmouth Junction, USA) in RPMI 1640 medium (#SH30809.01; Hyclone Laboratories LLC, Logan, UT, USA) without serum in the dark for 15 min under growth conditions. The cells were washed twice in RPMI medium containing 10% fetal bovine serum, centrifuged for 10 min at 400 × *g*, then were resuspended in sterile saline solution and counted; finally, they were diluted to 5 × 10^6^ cells per 1 ml. B7-H4 pregnant mice on Gd 8 were randomly divided into group 1, group 2 and group 3. Group 1: B7-H4 infected mice, Group 2: B7-H4 infected mice transferred with B7-H4 macrophages, and Group 3: B7-H4 infected mice transferred with WT macrophages. Then all the three groups were infected with 200 RH tachyzoites of *T. gondii*. Concomitantly, the infected B7-H4^-/-^ pregnant mice of group 1 were injected intravenously with 200 µl sterile saline solution, and the infected B7-H4^-/-^ pregnant mice of group 2 and group 3 were transferred intravenously with 1 × 10^6^ freshly isolated dMφ cells from B7-H4^-/-^ pregnant mice and WT pregnant mice, respectively. Pregnant mice in the three groups were sacrificed by cervical dislocation on Gd 14. The pregnancy outcome was observed, and monocytes were isolated and analyzed by flow cytometry.

### Collection of human clinical sample

Decidual tissues were obtained from healthy pregnant women who underwent voluntary abortion without any abortifacient or pregnancy complications in their first trimester (gestational age: 6–8 weeks). The sample collection for this study was approved by the Ethics Committee of Binzhou Medical University, and all women were patients of the Department of Obstetrics and Gynecology, Yantai Affiliated Hospital of Binzhou Medical University, Zhifu District Maternal and Child Health Hospital or Yantai Cancer Hospital. The samples were washed immediately 5–8 times in a sterile saline solution and stored in Dulbecco’s Modified Eagle’s Medium/high-glucose medium (#12100046; Hyclone Laboratories LLC) supplemented with 100 IU/ml penicillin/streptomycin.

### Preparation of human single cell

The decidual tissues were separated and cut into pieces, then transferred to a tissue breaking tube of the single-cell preparation apparatus (Miltenyi Biotec). Tissues were digested with 0.1% collagenase type IV (#C4-BIOC; Sigma-Aldrich, St. Louis, MO, USA) and 25 IU/ml DNase-I (#10104159001; Sigma-Aldrich) in incubators at 37 °C for 30 min . The resulting suspension was filtered through 75-µm nylon mesh filters. Mononuclear cells were isolated via density gradient centrifugation using human lymphocyte separation medium (#LTS1007; TBD Science, China) at 400 × *g* for 20 min at 20 °C in accordance with the manufacturer’s instructions. Approximately 3 × 10^7^ human decidual PBMCs were obtained and equally divided into uninfected, infected and B7-H4-neutralized infected groups. The mononuclear cells were incubated with 10 µg/ml anti-B7-H4 monoclonal antibody (mAb; #16–5949-82; Thermo Fisher Scientific) in the B7-H4-neutralized infected group for 2 h. *Toxoplasma gondii* tachyzoites were added to the infected group and to the B7-H4-neutralized infected group at a 2:1 ratio (*T. gondii*: cells). All study samples were cultured in RPMI 1640 medium supplemented with 10% FBS (Gibco, Thermo Fisher Scientific), 100 IU/ml streptomycin and 100 IU/ml penicillin for 20 h.

### Isolation of human decidual macrophages

Decidual macrophages were purified from the human mononuclear cells using the Human CD14 Positive Selection Kit (#17858; Stemcell Technologies, Vancouver, BC, Canada) following the manufacturer’s instructions, resulting in purity levels of > 95% (Additional file 2: Figure S1a, b). Approximately 3 × 10^6^ purified human CD14^+^ dMφ were divided equally into uninfected, infected and B7-H4-neutralized infected groups. In the B7-H4-neutralized infected group, dMφ were incubated with 10 mg/ml anti-B7-H4 mAb for 2 h before *T. gondii* tachyzoites were added at a 2:1 ratio (*T. gondii*: cells). Purified human primary CD14^+^ dMφ were equally divided into five groups: (i) uninfected; (ii) infected; (iii) STAT1-inhibitor infected; (iv) B7-H4-neutralized infected; and (v) B7-H4-neutralized STAT1-inhibitor infected group. dMφ of the STAT1-inhibitor infected group and B7-H4-neutralized STAT1-inhibitor infected group were preincubated with the STAT1 inhibitor fuldarabine (#HY-B0069; MedChemExpress) for 2 h, followed by addition of B7-H4 mAb. All study samples were cultured in RPMI 1640 medium supplemented with 10% FBS (Gibco, Thermo Fisher Scientific), 100 IU/ml streptomycin and 100 IU/ml penicillin for 20 h.

### Phagocytosis assay

Human CD14^+^ dMφ were suspended at a concentration of 1 × 10^6^ in culture medium, and 300 µl cells of uninfected, infected and B7-H4-neutralized infected groups were placed into a 24-well plate, respectively. The cells were incubated with 10 µg/ml anti-B7-H4 mAb in B7-H4-neutralized infected group for 2 h. *Toxoplasma gondii* tachyzoites were added to the infected and B7-H4-neutralized infected groups at a 1:1 ratio (*T. gondii*: cells). After 20 h of culture, rabbit IgG-FITC complex latex beads (#500290; Cayman Chemical Co., Ann Arbor, MI, USA) were mixed with macrophages at 37 °C for 2 h. Cells were incubated for 1 min with trypan blue quenching solution and washed with assay buffer at 4 °C, following which the phagocytic activity of macrophages was photographed under the fluorescence microscope and analyzed by flow cytometry.

### Flow cytometry

The purified human mononuclear cells were stained with the fluorescent intercalator 7-aminoactinomycin D (7-AAD) (#KGA219; KeyGEN NioTECH, Nanjing, China) and the following fluorochrome-conjugated mAbs: PE-cy7-conjugated anti-CD14 and APC-conjugated anti-B7-H4; FITC-conjugated anti-CD80, PE-conjugated anti-CD86, FITC-conjugated anti-CD206, FITC-conjugated anti-CD163. The mouse mononuclear cells were stained with the following mouse-specific mAbs: PE-cy7-conjugated anti-F4/80, PE-conjugated anti-CD206 and FITC-conjugated anti-TNF-α; APC-conjugated anti-B7-H4, FITC-conjugated anti-CD80, PE-conjugated anti-CD80 and APC-conjugated anti-iNOS; PE-conjugated anti-CD86, PE-conjugated anti-IL-10 and APC-conjugated anti-Arg-1. The live rate of mouse macrophages was > 95% (Additional file 2: Figure S1c), so we did not stain mouse mononuclear cells with 7-AAD. The human or mouse decidual lymphocytes were incubated with their corresponding mAbs at 4 °C in the dark for 30 min and then washed once. Cells were first incubated with antibodies against the cell surface proteins B7-H4, CD80, CD86, CD206, CD163 (human) and F4/80 (mouse) or CD14 (human), then fixed and permeabilized in 1 × Fix/Perm buffer (#00–5523-00; Thermo Fisher Scientific) for 30 min at 4 °C in accordance with the manufacturers’ protocol and washed twice; the cells were then incubated with antibodies of intracellular proteins (Arg-1, iNOS, IL-10 or TNF-α) at 4 °C in the dark for 40 min and washed once. To analyze for cytokines, the mononuclear cells were cultured for 4–6 h in a leukocyte activation cocktail (#51-20421E; eBioscience, San Diego, CA, USA) before adding the mAbs of cytokines. Analysis was performed using the BD FACSCanto™ II Flow Cytometer (BD, Franklin Lakes, NJ, USA).

### Western blotting

The CD14^+^ dMφ from each group was incubated for 24 h and lysed using ice-cold radioimmunoprecipitation lysis buffer (#P0013B; Beyotime, Shanghai, China) and phenylmethanesulfonyl fluoride (PMSF; #ST506-2; Beyotime) on ice for 40 min and then centrifuged for 20 min at 12,000 × *g*, 4 °C to remove the debris. The concentration of protein extracts was determined using a bicinchoninic acid protein assay kit (#PC00020; Solarbio) and boiled for 8 min in 5× sodium dodecyl sulfate-polyacrylamide gel electrophoresis (SDS-PAGE) sample loading buffer (#P0015; Beyotime). The total protein (30 µg) was separated by 12% SDS-PAGE (#P0012AC; Beyotime) and transferred to polyvinylidene difluoride (PVDF) membranes (#ISEQ00010; MilliporeSigma [formerly Merck Millipore], Burlington, MA, USA). The membranes were blocked for 2.5 h in 5% non-fat dry milk in Tris-buffered saline with Tween-20 at room temperature (20–25 °C), and then incubated overnight at 4 °C with the primary antibodies for B7-H4 (1:2000; Abcam, Cambridge, UK), CD80 (1:1000; Proteintech, Wuhan, China), Arg-1 (1:1000; Proteintech), iNOS (1:1000; Abcam), JAK2 (1:1000; Abcam), phosphorylated JAK2 (p-JAK2; 1:1000; Abcam), STAT1 (1:2000; Proteintech) and phosphorylated STAT1 (p-STAT1; 1:2000; Proteintech), glyceraldehyde-3-phosphate dehydrogenase (GAPDH; 1:40,000; Proteintech). The membranes were incubated with the appropriate secondary antibodies for 2 h at room temperature, and electroluminescence was detected using an enhanced chemiluminescence kit (#WLA006c; Wanleibio, China). Protein expression levels were determined using ImageJ software, and GAPDH was used as the internal control.

### Immunofluorescence

Purified human CD14^+^ dMφ from uninfected, infected and B7-H4-neutralized infected groups was air-dried onto polysine microscope adhesion slides. After fixation in 4% paraformaldehyde for 30 min, the slides were then blocked with goat serum for 1 h at room temperature. The cells were then incubated overnight at 4 °C with anti-B7-H4 (1:200; Abcam), anti-IL-10 (1:200; Abcam) and anti-TNF-α (1:200; Proteintech). After washing 3 times with PBS, the cells were incubated with the appropriate concentrations of secondary antibodies for 1 h at 37 °C. Subsequently, the cells were stained with the nucleic acid stain 4,6-diamidino-2-phenylindole (DAPI) for 15 min and washed once. Finally, cells were observed under a Zeiss LSM880 confocal microscope (Carl Zeiss AG).

## Results

### Establishment of the WT and B7-H4^-/-^ mouse models of adverse pregnancy caused by* T. gondii* infection

**Fig. 1:**
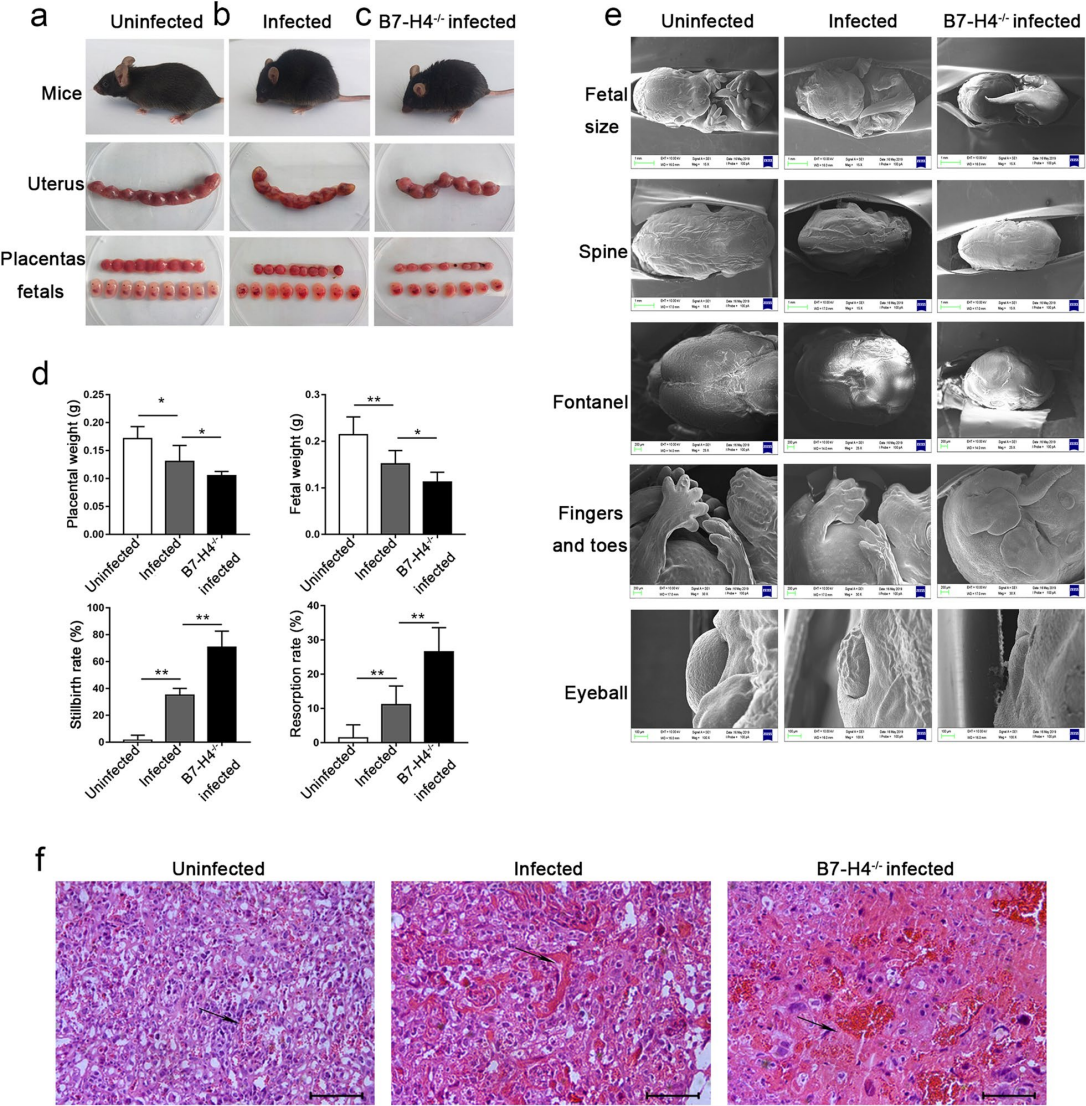
Effects of the inhibitory molecule B7-H4 on pregnancy outcomes during *Toxoplasma gondii* infection in mice. **a-c** Pregnancy outcomes in terms of mice, uterus, placenta and fetus in uninfected, infected and B7-H4^-/-^ infected groups. **d** Placental and fetal weight, stillbirth rates and resorption rates were analyzed in uninfected, infected and B7-H4^-/-^ infected groups. **e** Scanning electron microscopy (SEM) showing the different fetal development situations in the uninfected, infected and B7-H4^-/-^ infected groups. **f** Hematoxylin–eosin (HE) staining of uninfected, infected and B7-H4^-/-^ infected mouse placentas. Obvious hemorrhaging are shown by arrows. Scale bar: 100 µm. Data are presented as the mean ± standard deviation (SD), with at least 8 pregnant mice in each group assayed individually by unpaired t-test. Asterisks indicate significant differences between groups at *P < 0.05, **P < 0.01. B7-H4^-/-^, B7-H4 knockout

All mice were sacrificed and dissected on Gd 14 to observe the pregnancy outcomes. Consistent with our previous results [15], we observed that the pregnant mice in the uninfected group were in a good mental state with bright-colored hair and that the placenta and fetuses were well developed (Fig. 1a). However, the pregnant mice in the infected group were listless, with dark-colored hair and had smaller placenta and fetuses (Fig. 1b). Moreover, compared with the *T. gondii*-infected WT pregnant mice, B7-H4^-/-^ pregnant mice with *T. gondii* infection appeared to be more unresponsive, were trembling and had stillborn and absorbed fetuses (Fig. 1c). In addition, B7-H4^-/-^ pregnant mice with *T. gondii* infection had lighter (weight) placentas and fetuses compared with their normal counterparts. The weight of infected B7-H4^-/-^ pregnant mice was also lower than that of the infected group. The number of stillbirths and aborted fetuses significantly increased in this group after infection and further exacerbated under the condition of B7-H4 knockout and *T. gondii* infection (Fig. 1d).

Scanning electron microscopy (SEM) showed that the developmental conditions of fetal mice were significantly different among the three groups. Compared with the uninfected group, the fetal mice of the infected group had dysplasia with reduced fetal volume, an underdeveloped spinal column, prematurely closed fontanelle and underdeveloped finger fins and eyeball. Further, the dysplasia of fetal mice in the B7-H4^-/-^ infected group was more serious (Fig. 1e) compared with that of the infected group. Similarly, the infected group displayed more marked placenta bleeding than the uninfected group. Moreover, the placental bleeding of the B7-H4^-/-^ infected group was much greather than that of the infected mice (Fig. 1f).

### B7-H4 expression on dMφ was reduced after* T. gondii *infection

**Fig. 2:**
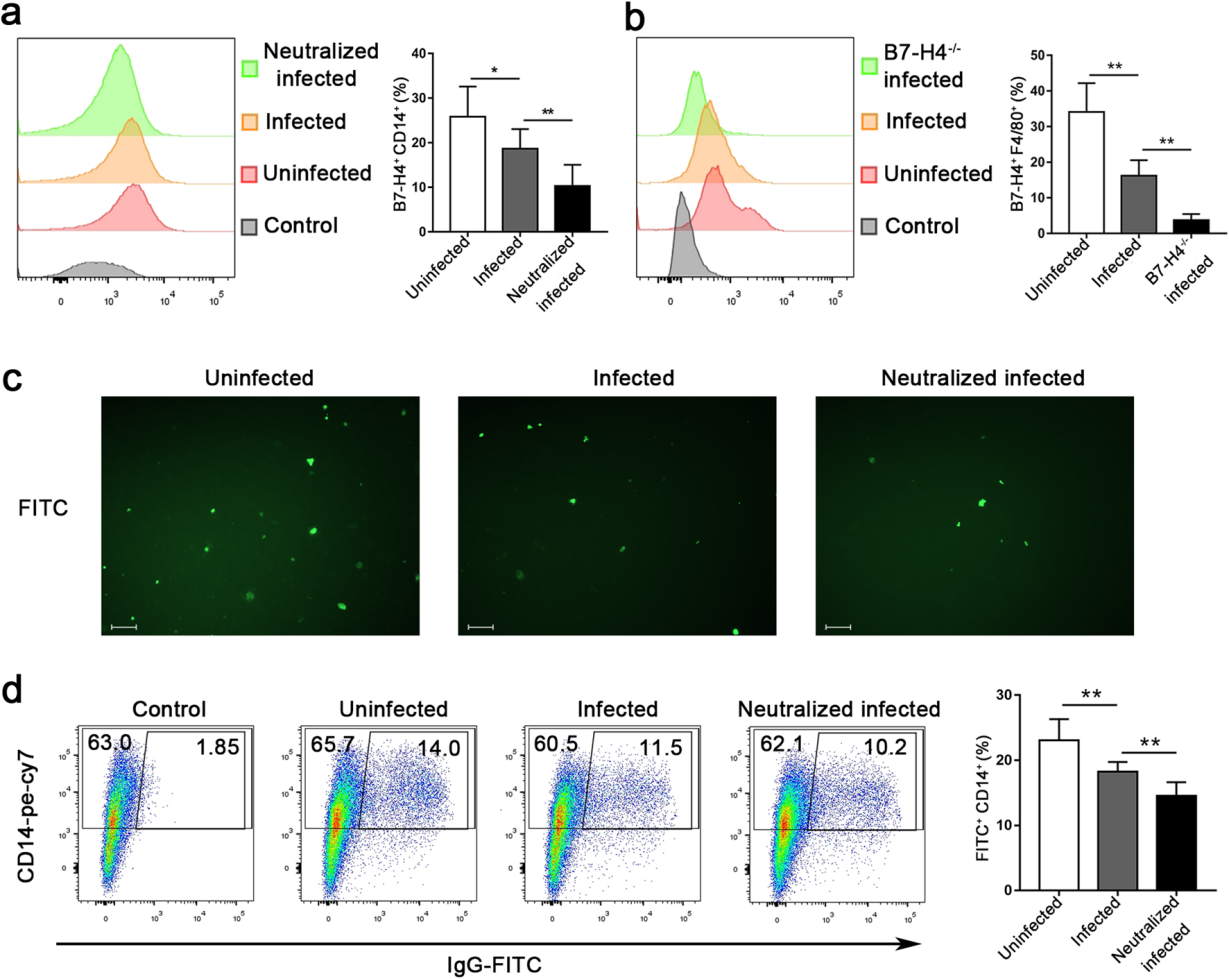
Changes in B7-H4 expression on decidual macrophages (dMφ) and the effect of these changes on phagocytic activities during *Toxoplasma gondii* infection. **a** B7-H4 expression levels on human CD14^+^ dMφ in uninfected, infected and B7-H4 neutralized infected groups detected by flow cytometry. **b** B7-H4 expression on mouse F4/80^+^ dMφ detected in uninfected, infected and B7-H4^-/-^ infected mice by flow cytometry. **c** Fluorescence microscopy images of the number of phagocytic dMφ in the uninfected, infected and B7-H4 neutralized infected groups. Scale bar: 50 µm. **d** Flow cytometry results showing the phagocytotic capacity of dMφ in the uninfected, infected and B7-H4 neutralized infected groups. Data are presented as mean ± SD, with at least 6 pregnant mice or human samples in each group assayed individually by the unpaired t-test. Asterisks indicated significant differences between groups at *P < 0.05, **P < 0.01. IgG-FITC, immunoglobulin G-fluorescein isothiocyanate complex

To explore the role of B7-H4 expression during *T. gondii* infection, B7-H4 expression levels on human CD14^+^ dMφ and mice F4/80^+^ dMφ were detected by flow cytometry. The results showed that the levels of B7-H4 expressed on human and mouse dMφ in the infected group were lower than those in the uninfected group. In addition, less B7-H4 was detected in B7-H4 neutralized human primary infected dMφ or in the B7-H4^-/-^ infected group compared to the infected group (Fig. 2a, b; Additional file 3: Figure S2a, b).

### Phagocytic ability of human dMφ declined with decreasing B7-H4 expression due to *T. gondii* infection

To further characterize the effect of B7-H4 downregulation on the function of dMφ, phagocytic activity was evaluated using fluorescein-labeled rabbit IgG-coated latex beads and detected by fluorescence microscopy and flow cytometry. The results showed that the FITC fluorescence phagocytosed by dMφ significantly decreased in the infected group compared with the uninfected group, while the phagocytosed FITC fluorescence was further decreased in the anti-B7-H4 neutralized infected group compared with the infected group (Fig. 2c, d; Additional file 3: Figure S2c), indicating that the decrease in B7-H4^-/-^ expression after *T. gondii* infection could weaken the phagocytosis ability of dMφ.

### B7-H4 downregulation by* T. gondii* infection affected the expression of M1/M2 membrane functional molecules

**Fig. 3:**
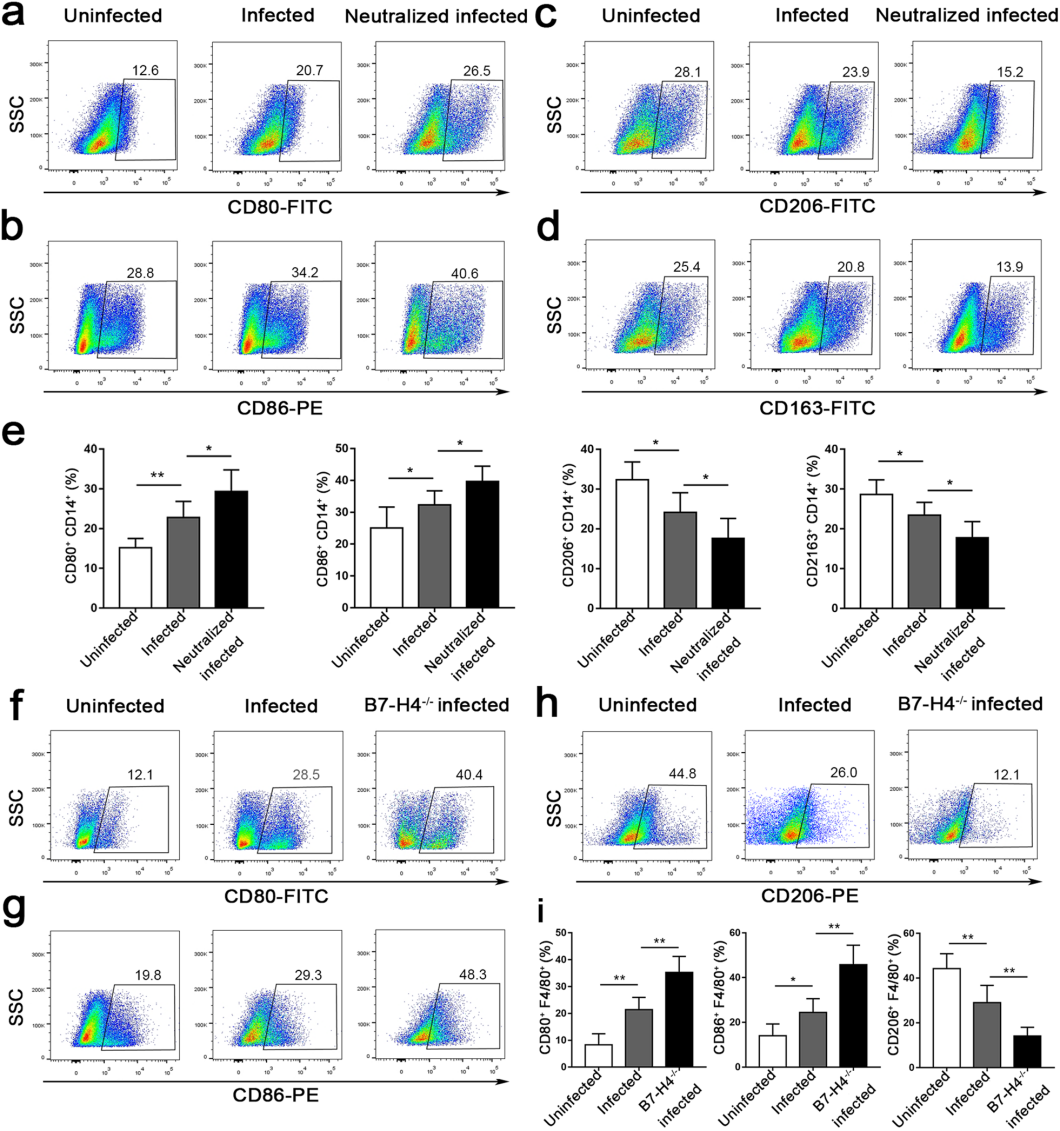
Reduction of B7-H4 expression on dMφ by *T. gondii* infection affects the expression of M1- and M2-type membrane functional molecules. **a-e** Flow cytometry analysis of CD80, CD86, CD206 and CD163 levels on human dMφ in uninfected, infected and B7-H4 neutralized infected groups. **f-i** Flow cytometry analysis of B7-H4, CD80, CD86 and CD206 expressions on dMφ in uninfected, infected, and B7-H4^-/-^ infected mice. Data are presented as the mean ± SD, with at least 6 pregnant mice or human samples in each group assayed individually by unpaired the t-test. Asterisks indicate significant differences between groups at *P < 0.05, **P < 0.01. PE, Phycoerythrin; SSC, side scatter

Flow cytometry was used to detect the levels of membrane molecules on the surface of human dMφ in the three groups of mice. Concomitant with the decrease in B7-H4 expression, the expression levels of the M1-type membrane molecules CD80 and CD86 were significantly higher in the infected group than in uninfected group. In the anti-B7-H4 neutralized infected group, the expression of M1-type membrane molecules were further upregulated compared with those in the infected group (Fig. 3a, b). However, levels of the M2-type membrane molecules CD163 and CD206 were significantly decreased after infection, and they were further downregulated in the B7-H4 neutralized infected group compared to the infected group (Fig. 3c, d).

Similar to the results in the vitro study, the expression levels of M1-type membrane molecules CD80 and CD86 were also significantly upregulated in the infected pregnant mice compared with the uninfected group, whereas the expression level of the M2-type membrane molecule CD206 was significantly decreased. Compared with the infected mice, the expression levels of M1-type membrane molecules CD80 and CD86 were further upregulated in the infected B7-H4^-/-^ pregnant mice, while

### B7-H4 reduction by* T. gondii* infection associated with the expression of Arg-1 and iNOS in dMφ

**Fig. 4:**
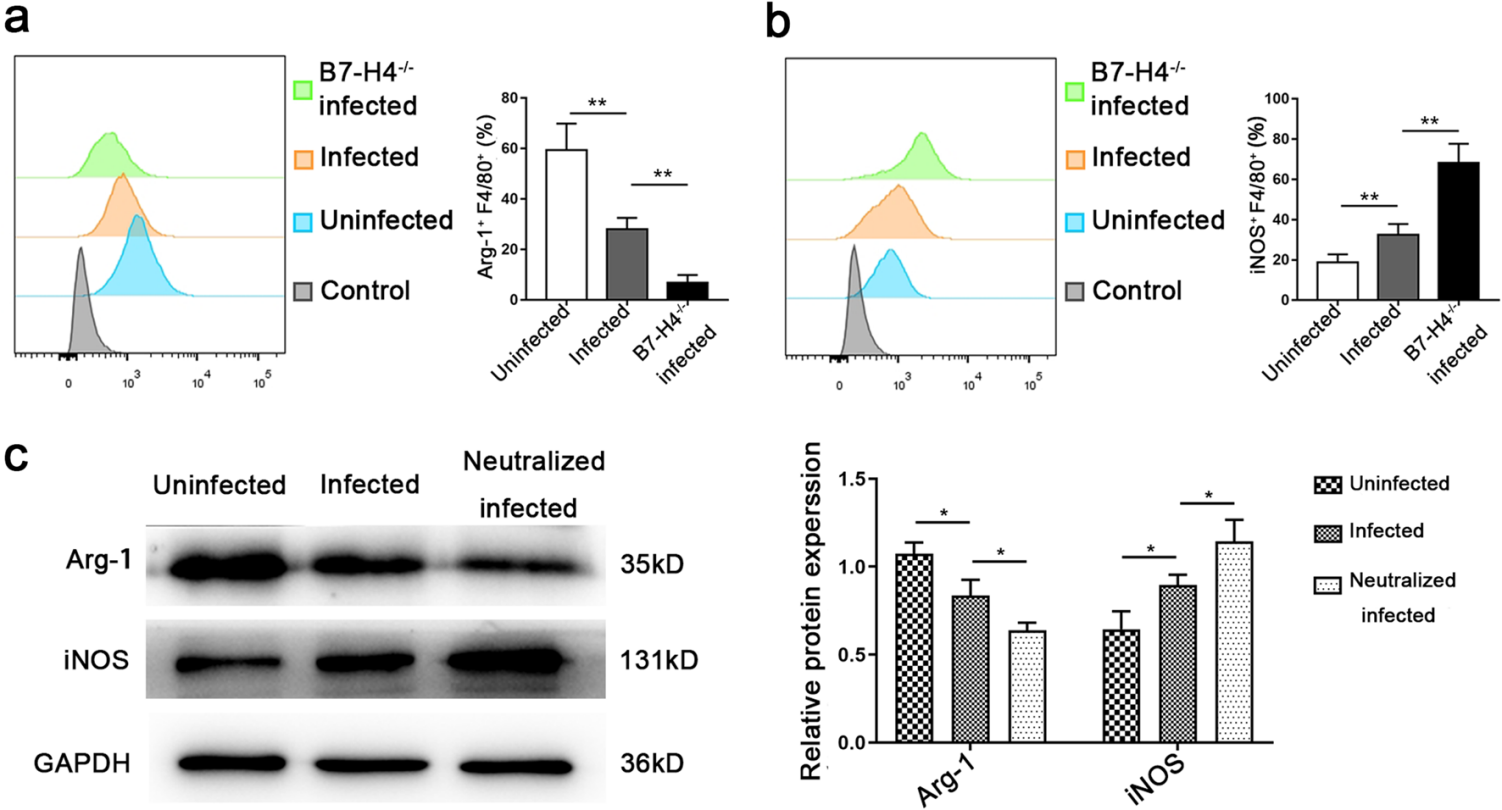
Reduction of B7-H4 expression on dMφ with *T. gondii* infection resulted in changes in the expression iNOS and Arg-1. **a** Levels of Arg-1 produced by dMφ in uninfected, infected and B7-H4^-/-^ infected mice analyzed by flow cytometry. **b** Flow cytometry analysis of iNOS produced by dMφ in uninfected, infected, and B7-H4^-/-^ infected mice. **c** Western blotting assay of B7-H4, iNOS and Arg-1 protein levels in uninfected, infected and B7-H4 neutralized infected groups of purified human macrophages. Data are presented as the mean ± SD, with at least 6 pregnant mice or human samples in each group assayed individually by the unpaired t-test. Asterisks indicate significant differences between groups at *P < 0.05, **P < 0.01. Arg-1, Arginase-1; GAPDH, glyceraldehyde-3-phosphate dehydrogenase; iNOS, inducible nitric oxide synthase

The expressions of the arginine catabolism enzyme (Arg-1) and iNOS in dMφ in the uninfected, infected and B7-H4^-/-^ infected groups were detected by flow cytometry. The results showed that the expression of iNOS increased in dMφ of infected mice while that of Arg-1 decreased. Moreover, the expression of iNOS was further upregulated and that Arg-1 was further downregulated in dMφ of the B7-H4^-/-^ infected group compared with the infected group (Fig. 4a, b).

In vitro, the expression of iNOS and Arg-1 in human dMφ were detected by western blotting. The results showed that iNOS was clearly upregulated after *T. gondii* infection, while Arg-1 was significantly downregulated. After being treated by B7-H4 neutralized antibody, the synthesis of iNOS in the infected human dMφ was further upregulated, whereas that of Arg-1 was further downregulated, compared with the infected group (Fig. 4c).

### B7-H4 downregulation by* T. gondii* infection was related to the cytokines IL-10 and TNF-α produced by dMφ

**Fig. 5:**
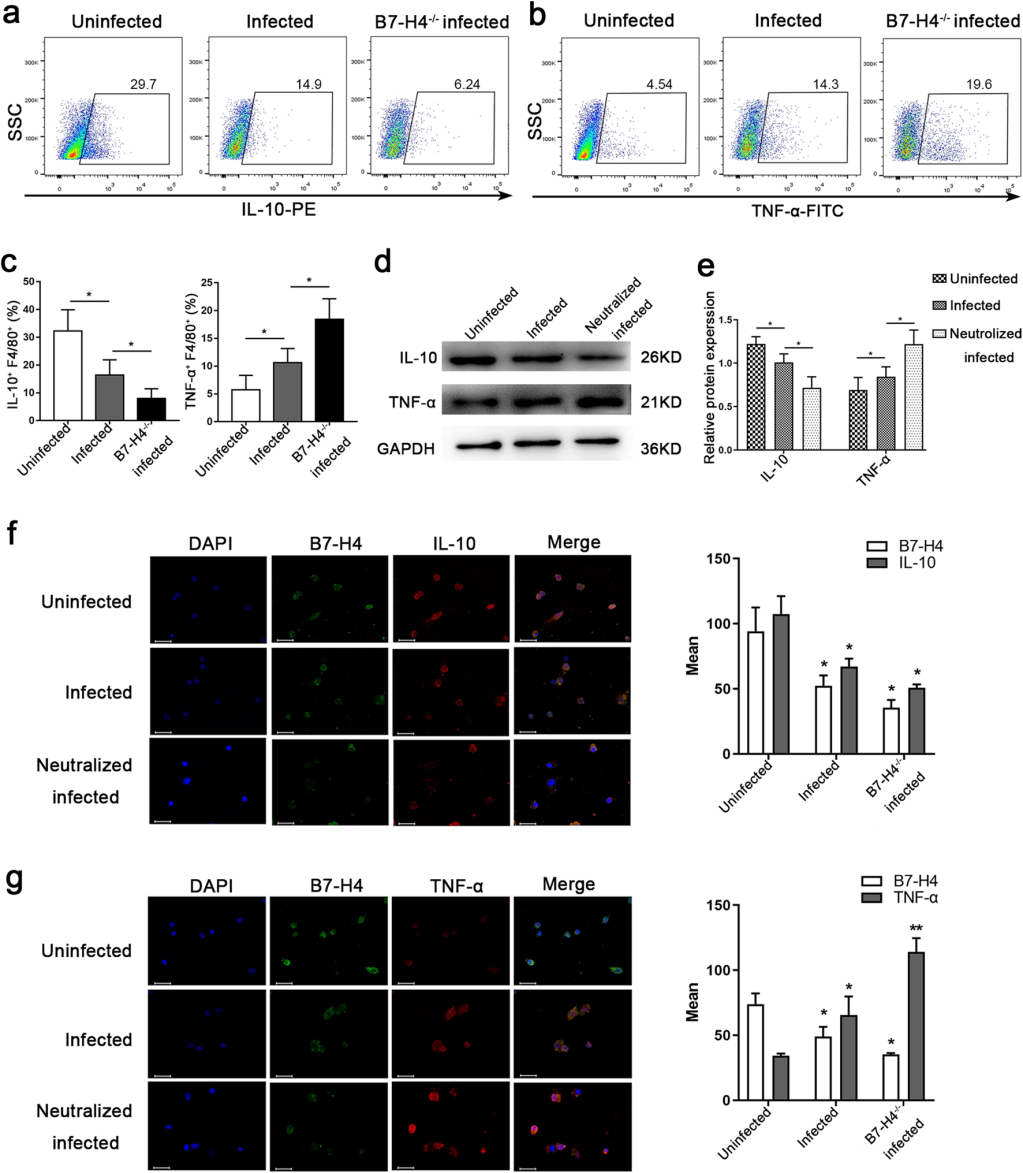
Reduction of B7-H4 expression on dMφ with *T. gondii* infection resulted in changes in the expression of TNF-α and IL-10. **a-c** Expression level of IL-10 and TNF-α in F4/80^+^ dMφ in uninfected, infected and B7-H4^-/-^ infected mice detected by flow cytometry. **d**, **e** Western blotting assay of TNF-α and IL-10 levels in CD14^+^ dMφ of the uninfected, infected and B7-H4 neutralized infected groups. **f**, **g** Representative immunofluorescent photographs of B7-H4, TNF-α and IL-10 of CD14^+^ dMφ in the uninfected, infected and B7-H4 neutralized infected groups. Scale bar: 50 µm. Data are presented as the mean ± SD, with at least 6 pregnant mice or human samples in each group assayed individually by the unpaired t-test. Asterisks indicate significant differences between groups at *P < 0.05, **P < 0.01. DAPI, 4′,6-Diamidino-2-phenylindole; IL, interleukin; TNF, tumor necrosis factor

The levels of the M2-type macrophage-associated cytokine IL-10 and M1-type macrophage-associated cytokine TNF-α were detected both in vitro and in vivo. Flow cytometry results showed that the IL-10 produced by dMφ was lower in B7-H4^-/-^ infected pregnant mice (Fig. 5a) but that TNF-α levels were higher than those in the infected group (Fig. 5b). In vitro, the expression of IL-10 and TNF-α in human dMφ were detected by western blotting and immunofluorescence. The results showed that the production of TNF-α was increased after *T. gondii* infection, while IL-10 production was significantly decreased. In *T. gondii*-infected B7-H4-neutralized human dMφ, TNF-α was further upregulated, whereas IL-10 was further downregulated, compared with the infected group (Fig. 5d-g).

### B7-H4 regulated the iNOS and TNF-α expression of dMφ through the JAK2/STAT1 signaling pathways

**Fig. 6:**
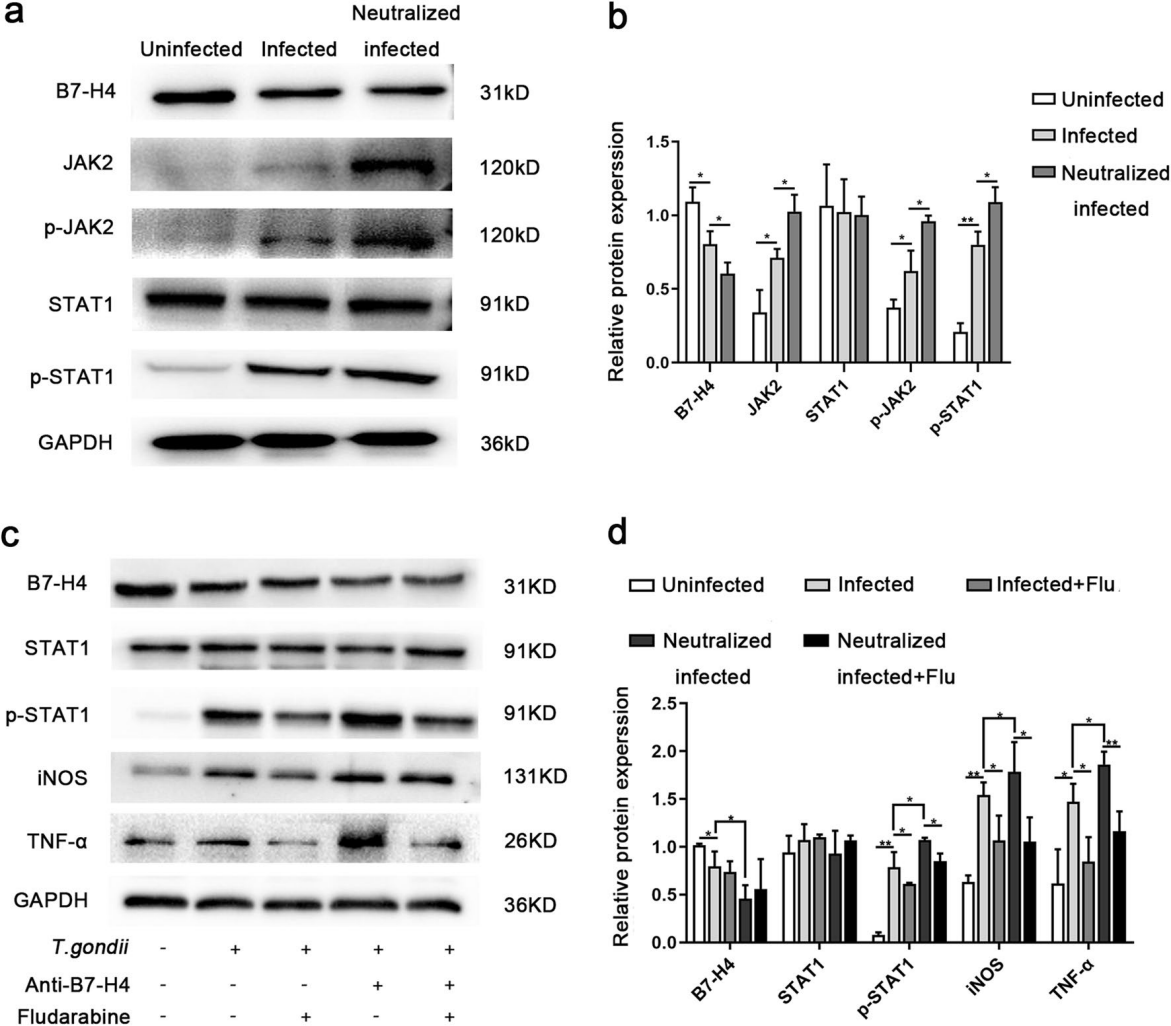
Downregulation of B7-H4 by *T. gondii* infection resulted in changes in dMφ function by affecting the JAK2/STAT1 signaling pathways. **a**, **b** Representative western blot and histogram analysis of B7-H4, JAK2, p-JAK2, STAT1 and p-STAT1 levels of dMφ in uninfected, infected and B7-H4 neutralized infected groups. **c**, **d** Representative western blot and histograms analysis of B7-H4, STAT1, p-STAT1, iNOS and TNF-α of dMφ in the uninfected, infected, infected + STAT1 inhibitor (Infected + Flu), B7-H4 neutralized infected and B7-H4 neutralized infected + STAT1 inhibitor (Neutralized infected + Flu) groups. The data in all panels are representative of at least three independent experiments. Data are presented as the means ± SD, with the unpaired t-test. Asterisks indicate significant differences between groups at *P < 0.05, **P < 0.01. Flu, Fludarabine; JAK2, Janus kinase 2; p-JAK2, phosphorylated JAK2; p-STAT1, phosphorylated signal transducer and activator of transcription 1; STAT1, signal transducer and activator of transcription 1

The B7-H4 down regulation after *T. gondii* infection increased iNOS and TNF-α expression through JAK2/STAT1 signaling pathway. In order to investigate the specific mechanism by which the downregulation of B7-H4 in human early pregnancy regulated the function of dMφ, the expression of several key molecules of the JAK2/STAT1 signaling pathway were examined by Western blotting. The results showed that as the dMφ B7-H4 decreased after *T. gondii* infection, the expressions of JAK2, p-JAK2, p-STAT1, iNOS and TNF-α increased, and the expression of these key molecules were more significantly up regulated after a large margin B7-H4 decrease caused by B7-H4 neutralized antibody treatment (Fig. 6a, b). Western blotting also showed that there was no significant change in B7-H4 level of *T. gondii*-infected dMφ after treated by STAT1 inhibitor compared with the uninhibited groups. Interestingly, the expression levels of iNOS and TNF-α were decreased under STAT1 inhibited in *T. gondii*-infected dMφ (Fig. 6c, d).

### Adoptive transfer of dMφ from WT mice could restore the dMφ dysfunction caused by* T. gondii* infection and alleviate the adverse pregnancy outcomes

**Fig. 7:**
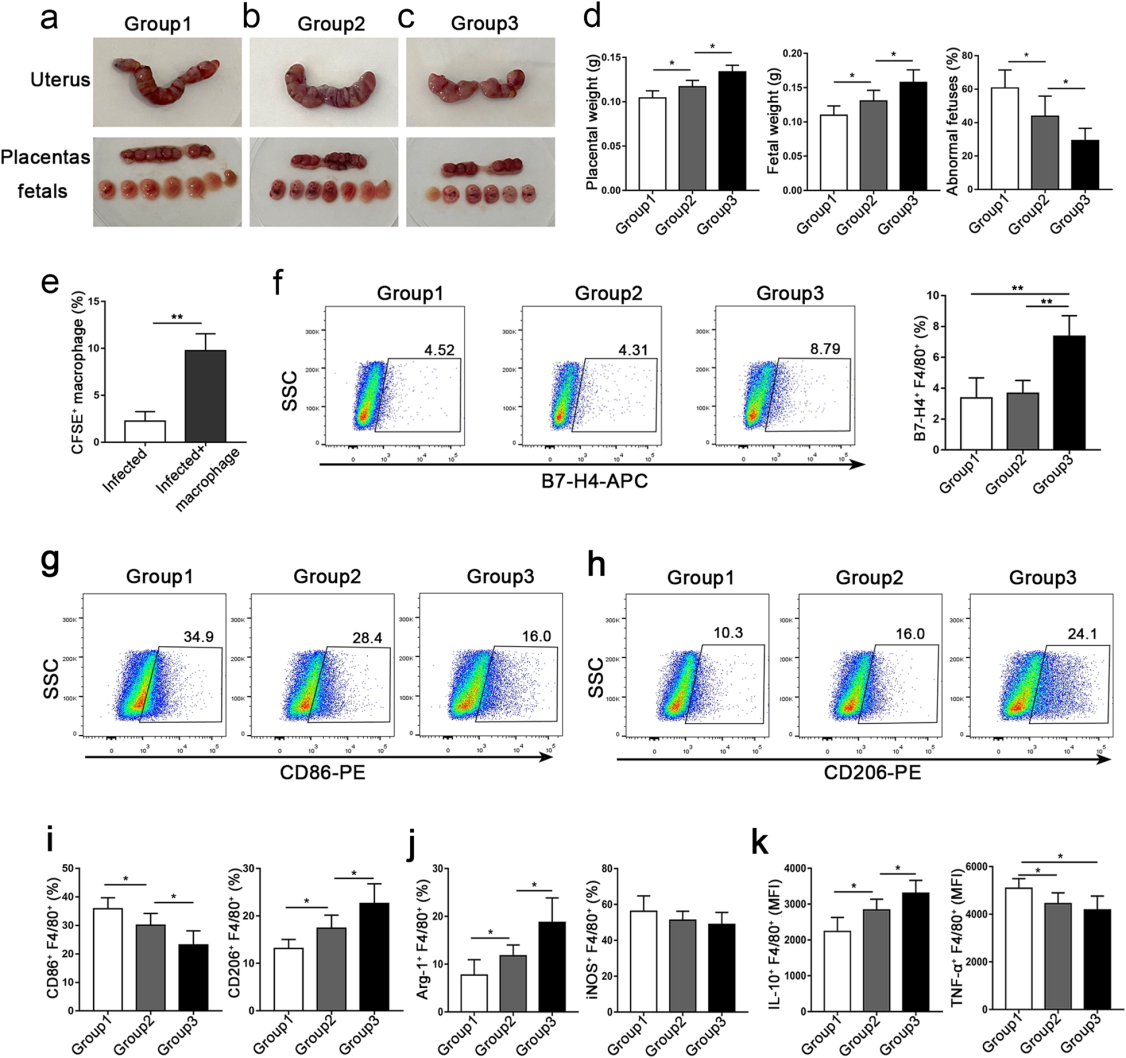
Adoptive transfer of dMφ from wild-type mice alleviated the adverse pregnancy outcomes and reversed the dysfunction of dMφ caused by *T. gondii* infection. **a-c** Pregnancy outcomes: fetuses and placentas in the three groups. **d** Comparison of placenta, fetal weight and rate of abnormal fetuses in pregnant mice in the three groups. **e** Proportion of CFSE^+^ macrophages in the placenta of transplanted mice. **f** Level of B7-H4 on the surface of dMφ in the three groups. **g**, **h** Flow cytometry was used to detect the expression of CD86 and CD206 in the three groups. **i** Detection of the expressions of Arg-1 and iNOS in the three groups by flow cytometry. **j**, **k** Detection of the expressions of IL-10 and TNF-α in the three groups by flow cytometry. Group 1, B7-H4^-/-^ infected mice; group 2, B7-H4^-/-^ infected mice after adoptive transfer of B7-H4^-/-^ dMφ; group 3: B7-H4^-/-^ infected mice after the adoptive transfer of WT dMφ. Data are presented as the mean ± SD, with at least 6 pregnant mice or human samples in each group assayed individually by the unpaired t-test. Asterisks indicate a significant difference between groups at *P < 0.05, **P < 0.01

To further confirm whether the adverse pregnancy outcomes caused by *T. gondii* infection were due to the decrease of B7-H4 on dMφ, the dMφ from WT or B7-H4^-/-^ mice were respectively adoptively transferred to B7-H4^-/-^ mice with adverse pregnancy outcomes caused by *T. gondii* infection. After the adoptive transfer of dMφ from B7-H4^-/-^ mice, there was a slight increase in placental and fetal volumes, whereas there was a minimal decrease in the abnormal fetal rate (Fig. 7a, b, d). In addition, placental and fetal volumes were significantly increased and the abnormal fetal rate was greatly reduced following the adoptive transfer of dMφ from WT mice (Fig. 7b–d). The dMφ from donor WT or B7-H4^-/-^ pregnant mice that reached the recipient placenta accounted for approximately 10% of the dMφ in transfused mice (Fig. 7e). In addition, the B7-H4 levels on the dMφ of the infected B7-H4^-/-^ pregnant mice were significantly upregulated after the adoptive transfer of WT dMφ, but no differential change was observed in the B7-H4^-/-^ dMφ donor group (Fig. 7f). To determine whether the dysfunctions of dMφ were related to the downregulation of B7-H4 caused by *T. gondii* infection, the functional molecules of dMφ were analyzed by flow cytometry. After adoptive transfer of dMφ from WT mice to *T. gondii*-infected B7-H4^-/-^ mice, the expression level of the M1-type membrane molecule CD86 greatly decreased in the recipient dMφ, whereas that of the M2-type membrane molecular CD206 notably increased. However, the expression level of CD86 slightly decreased and CD206 showed even a lower increase after the adoptive transfer of dMφ from B7-H4^-/-^ mice (Fig. 7g–i). The expression level of Arg-1 was significantly upregulated after the transfer of dMφ from WT mice but was only slightly upregulated after the transfer from B7-H4^-/-^ mice, and iNOS expression showed no detectable change after the transfer (Fig. 7j). The expression level of IL-10 was significantly upregulated after accepting the transfer of dMφ from WT mice but only slightly upregulated after that from B7-H4^-/-^ mice, while the expression level of TNF-α decreased after the transfer of dMφ from WT or B7-H4^-/-^ mice (Fig. 7k).

## Discussion

*Toxoplasma gondii* is an important opportunistic protozoan, and infection can disrupt the immune microenvironment at the maternal–fetal interface during early pregnancy, leading to adverse pregnancy outcomes [28]. At the maternal–fetal interface, an appropriate immune microenvironment is essential for a successful pregnancy [29]. dMφ play an important role in successful pregnancies as the second largest immune cell [30]. An increasing number of studies have shown that dMφ promote immune tolerance during normal pregnancy, which is similar to the function of M2-type macrophages [31]. B7-H4, as a negative immunomodulatory molecule, is consistently highly expressed on dMφ and plays an important immunosuppressive role in maintaining maternal–fetal tolerance of a normal pregnancy [14, 18]. In addition, the level of B7-H4 in pregnant women with an abnormal pregnancy is lower than that in women with a normal pregnancy [32]. However, whether the level of B7-H4 on dMφ changes after *T. gondii* infection and whether the changes of B7-H4 on dMφ are related to adverse pregnancy outcomes need to be further explored. In the present study, B7-H4^-/-^ infected pregnant mouse models were established and the pregnancy outcomes were observed. The results showed that the adverse pregnancy outcomes were more severe in *T. gondii*-infected B7-H4^-/-^ pregnant mice than in *T. gondii*-infected WT mice, indicating that the change in B7-H4 level may play an important role in abnormal pregnancy outcomes induced by *T. gondii* infection. Furthermore, the expression level of B7-H4 on dMφ was downregulated in *T. gondii*-infected mice compared with the uninfected group. The same result in vitro was obtained when using human primary dMφ with *T. gondii* infection. However, whether the B7-H4 downregulation on dMφ after *T. gondii* infection could induce dMφ dysfunction and the related mechanism are still unclear.

The phagocytic activity of macrophages is an important marker for assessing their polarization direction: M1-type macrophages mainly promote T helper type 1 (Th1) response by producing cytokines, while M2-type macrophages have strong phagocytic activity and mediate T helper type 2 (Th2) response, promote tissue repair and produce immunosuppression [33, 34]. Our results showed that the phagocytic activity of dMφ was reduced after *T. gondii* infection. After the B7-H4 function was blocked with anti-B7-H4 neutralized antibody, the phagocytic activity of human dMφ further decreased compared with that of the infected cells. These data suggest that the downregulation of B7-H4 can result in polarization of dMφ from M2-type to M1-type macrophages. The dMφ present in the pregnant uterus or decidual are a heterogeneous population, and the majority of dMφ have the M2 phenotype with high levels of membrane molecules (CD206 and CD163) [35]. The M2-phenotype macrophages has immunosuppressive properties that help in tissue remodeling and promoting Th2 immune responses [36]. The M1-phenotype macrophages have antigen-presentation ability and promote the development of inflammation, with high expression of CD80 and CD86 [37]. To further explore whether decreased B7-H4 expression can regulate the expression level of M1/M2 membrane molecules on dMφ, we used flow cytometry to detect the membrane molecules in vitro and in vivo. The results showed that the levels of the M1-type membrane molecules CD80 and CD86 were increased on human dMφ after infection with *T. gondii*, while the levels of the M2-type membrane molecules CD206 and CD163 were decreased. To further explore the changes of the above-mentioned membrane molecules caused by the decreased B7-H4, B7-H4 neutralized antibody was used to block the function of B7-H4 in the infected human dMφ in vitro and the B7-H4^-/-^ infected pregnancy mouse model was used. The results showed that the expression levels of the M1-type membrane functional molecules CD80 and CD86 increased and those of the M2-type membrane functional molecules CD163 and CD206 decreased, in the B7-H4 neutralized infected group and B7-H4^-/-^ infected pregnant mice compared with the infected group. These results revealed that the downregulation of B7-H4 on dMφ induced by *T. gondii* infection could promote the expression of M1-type membrane molecules and decreased that of M2-type membrane molecules. As a result, the immune tolerance function of M2-type dMφ would be weakened, which would induce the dysfunction of dMφ and contribute to abnormal pregnancy outcomes.

The immune tolerance function of macrophages is also related to the expression of several intracellular enzymes, such as Arg-1 and iNOS, which are involved in the inflammatory response and exhibit different immune regulation effects [38]. Arg-1 can be used as a marker of M2-phenotype macrophages. Any increase in the synthesis or activity of Arg-1 can reduce the synthesis of NO, which participates in the maintenance of maternal–fetal tolerance [39]. Conversely, iNOS is barely expressed during a normal pregnancy, and its excessive production can promote the synthesis of NO, which may give rise to early embryo loss [40]. The results of the present study showed that the expression level of iNOS significantly increased in dMφ after infection with *T. gondii*, both* in vitro and in vivo*, while the expression of Arg-1 decreased. To determine whether the changes in iNOS and Arg-1 expression were induced by B7-H4 downregulation, we examined the levels of iNOS and Arg-1 in the *T. gondii*-infected anti-B7-H4 neutralized human dMφ and *T. gondii*-infected B7-H4^-/-^ mice. The results showed that compared with the infected groups, the production of iNOS increased, but that of Arg-1 decreased. These results indicate that the downregulation of B7-H4 on dMφ induced by *T. gondii* infection can dysregulate the synthesis of arginine metabolism-related enzymes and result in the dysfunction of dMφ due to shifting M2-type macrophages toward the M1 phenotype, which may also eventually contribute to adverse pregnancy outcomes.

Besides, dMφ can also secrete numerous cytokines, such as IL-10 and TNF-α, which participate in dMφ function of maternal–fetal tolerance [41]. M1-type macrophages mainly secrete high level of TNF-α, whereas M2-type macrophages secrete more IL-10 than their M1 counterparts [42]. Tumor-associated macrophages exhibit a high and sustained expression of B7-H4 and secrete a large amount of IL-10 [43]. The expression level of B7-H4 is higher in M2-type macrophages than in M1-type macrophages in infiltrating ductal carcinoma tissues, and the secretion of IL-10 is positively correlated with the expression of B7-H4 [19]. Inversely, the secretion of TNF-α in salivary gland lymphocytes of mice was found to increase significantly after neutralization by the anti-B7-H4 monoclonal antibody [44]. Thus, the major cytokines, TNF-α and IL-10, produced by dMφ may result in the imbalance of M1 and M2 phenotypes caused by the downregulation of B7-H4. Interestingly, the results showed that the expression of cytokine IL-10 was downregulated and that of TNF-α was upregulated in *T. gondii*-infected B7-H4 neutralized human dMφ and *T. gondii*-infected B7-H4^-/-^ pregnant mice compared with the corresponding infected group. These results provide further evidence that the expression changes of TNF-α and IL-10 in our mouse model were related to B7-H4 downregulation on dMφ by *T. gondii* infection, which were associated with the dysfunction of dMφ and harmful to maternal–fetal immune tolerance.

These results indicate that the downregulation of B7-H4 on dMφ caused by *T. gondii* infection can influence the maternal–fetal tolerance function of dMφ and ultimately contribute to adverse pregnancy outcomes. However, how the decreased B7-H4 regulates the changes of the above-mentioned functional molecules requires further exploration.

 The level of the STAT1 gene has been shown to increase in CD34^+^HPC cells of B7-H4^-/-^ mice with human cytomegalovirus infection [21]. The results of one study suggest that STAT1 is an essential mediator of M1-type macrophage polarization [22]. Moreover, the expression of the M1-type functional molecules iNOS and TNF-α are reported to be regulated by the JAK2/STAT1 signaling pathway [23]. However, whether the expression of iNOS and TNF-α in dMφ is regulated by the downregulation of B7-H4 after *T. gondii* infection through JAK2/STAT1 signaling pathways still needs to be explored. In the present study, the key signal molecules of these signaling pathways were analyzed by western blotting, and the results showed that the expression level of JAK2 increased after *T. gondii* infection, as did the extent of phosphorylation of JAK2 and STAT1. We then used *T. gondii*-infected human B7-H4 neutralized dMφ to determine whether the changes in these signal molecules were associated with the downregulation of B7-H4. The results showed that the JAK2/STAT1 signal pathway was activated after the function of B7-H4 in the *T. gondii*-infected human dMφ was blocked. Furthermore, the levels of iNOS and TNF-α produced by *T. gondii*-infected dMφ were increased after B7-H4 neutralization, indicating that the reduced expression of B7-H4 may regulate iNOS and TNF-α through the JAK2-STAT1 signaling pathways. To further explore whether B7-H4 can regulate the expression levels of iNOS and TNF-α through the JAK2**/**STAT1 signaling pathways, we used STAT1 inhibitors in the infected group and B7-H4 neutralized infected group. Our data showed that after the inhibition of STAT1, the expression levels of iNOS and TNF-α fell in *T. gondii*-infected dMφ, irregardless of whether B7-H4 was neutralized or not. These results suggest that the immunosuppressive molecule B7-H4 could regulate the expression of iNOS and TNF-α in *T. gondii*-infected dMφ through the JAK**/**STAT1 signaling pathway, which plays a significant role in the maintenance of normal pregnancy.

Macrophages at the maternal–fetal interface are similar to M2-phenotype macrophages, and the adoptive transfer of M2-type macrophages can reduce the extent of inflammatory response in mice [45, 46]. dMφ expressing high levels of B7-H4 contribute to the maintenance of maternal–fetal immune tolerance during pregnancy [14]. In order to explore whether the downregulation of B7-H4 on dMφ induced by *T. gondii* infection contributes to an abnormal pregnancy, dMφ from WT or B7-H4 mice were respectively adoptively transferred to *T. gondii*-infected B7-H4^-/-^ pregnant mice. Our study showed that the pregnancy outcomes in *T. gondii*-infected B7-H4^-/-^ mice adoptively transferred with dMφ from WT mice were better than those from B7-H4 mice, indicating that the downregulation of B7-H4 on dMφ induced by *T. gondii* infection contributed to abnormal pregnancy. After the adoptive transfer of dMφ from WT mice, the B7-H4 level in the infected recipient B7-H4^-/-^ mice was upregulated, the levels of M1-related functional molecules CD86 and TNF-α were clearly decreased and the levels of the M2-related functional molecules (CD206, Arg-1, and IL-10) were all significantly increased. However, after the adoptive transfer of dMφ from B7-H4^-/-^ mice, the levels of CD86 and TNF-α in the recipient mice slightly decreased, while the levels of M2-related functional molecules (CD206, Arg-1, and IL-10) showed minimal increases. The level of the M1-related functional molecule iNOS showed no significantly change after the transfer from WT or B7-H4^-/-^ mice. These results suggest that along with the increased level of B7-H4 by the adoptive transfer of dMφ from WT mice, the immune tolerance function of maternal–fetal was enhanced, and the adverse pregnancy outcomes caused by *T. gondii* infection were alleviated. These results also demonstrated that the expression level of B7-H4 on dMφ plays an important role in an adverse pregnancy due to *T. gondii* infection.

## Conclusions

The results of this study demonstrate that the downregulation of B7-H4 on dMφ induced by *T. gondii* infection resulted in the abnormal expression of membrane molecules, synthesis of arginine metabolic enzymes and production of cytokines, all of which caused dMφ dysfunction and contributed to adverse pregnancy outcomes. The decrease in B7-H4 expression after *T. gondii* infection affected iNOS expression and TNF-α production through the JAK2/STAT1 signaling pathway, which weakened the maternal–fetal tolerance function due to dMφ polarization from M2-type macrophages toward to M1-type macrophages. The upregulation of B7-H4 by the adoptive transfer of dMφ was able to alleviate the adverse pregnancy outcomes caused by *T. gondii* infection. Our findings are of great value in improving our understanding of the molecular and immune mechanism of adverse pregnancy outcomes caused by *T. gondii* infection.

## Supplementary Information

**Additional file 1:** **Text S1. **Supplementary description of methods.

**Additional file 2:** **Figure S1.** The purity of human decidual macrophages. **a** Flow cytometry results showing the percentage of human decidual macrophages before purified by human CD14 positive selection kit. **b** Flow cytometry results showing the percentage of human decidual macrophages after purified by human CD14 positive selection kit. **c** The proportion of live cells in mouse.

**Additional file 3:** **Figure S2.** **a** Gating strategy for flow cytometry in the human experiment. **b** Gating strategy for flow cytometry in mice experiment. **c** Gating strategy for flow cytometry in phagocytosis assay experiment of human decidual macrophages.

## Data Availability

All data generated in this study are presented within this published article.

## Abbreviations

Arg-1: Arginase-1
CFSE: Carboxyfluorescein succinimidyl amino ester
DAPI: 4′,6-Diamidino-2-phenylindole
dMφ: Decidual macrophage
Gd: Gestational day
iNOS: Inducible nitric oxide synthase
IL: Interleukin
JAK2: Janus kinase 2
mAb: Monoclonal antibody
p-JAK2: Phosphorylated JAK2
p-STAT1: Phosphorylated STAT1
PBS: Phosphate-buffered saline
PMSF: Phenylmethylsulfonyl fluoride
PVDF: Polyvinylidene difluoride
SEM: Scanning electron microscopy
STAT1: Signal transducer and activator of transcription 1
TNF-α: Tumor necrosis factor-alpha
WT: Wild-type
