# Supplementary material for: Effect of B7-H4 downregulation induced by Toxoplasma gondii infection on dysfunction of decidual macrophages contributes to adverse pregnancy outcomes
Source: Parasit Vectors. 2022 Dec 13;15:464. doi: 10.1186/s13071-022-05560-9 (PMC9746109; doi:10.1186/s13071-022-05560-9)
Supplement: Supplementary file 1 — Additional file 1: Text S1. Supplementary description of methods. [file 13071_2022_5560_MOESM1_ESM.docx]

# Methods and Materials Ethics Statement

1. The sample collection procedures for this study were approved by the Binzhou Medical University Ethics Committee (Shandong, P. R. China).
2. All subjects provided a written informed consent for the collection of samples and the subsequent analysis.
3. This study was carried out in strict accordance with the recommendations in the Guide for the Care and Use of Laboratory Animals of Binzhou Medical University.
4. The protocol was approved by the Committee on the Ethics of Animal Experiments of Binzhou Medical University.
5. All procedures were performed under sodium pentobarbital anesthesia, and all efforts were exerted to minimize the suffering of animals.

# Experiment Reagent

Table 1. List of human flow antibodies

| Antibody | Cat.No | fluorescein | Con | Clone | Brand |
| --- | --- | --- | --- | --- | --- |
| anti-human CD14 | 25-0149-42 | PE-cy7 | 0.2mg/ml | 61D3 | eBioscience, USA |
| anti-human B7-H4 | 358107 | APC | 0.15mg/ml | MZH43 | Biolegend, USA |
| anti-human CD206 | 551135 | FITC | 0.05mg/ml | 19.2 | BD, USA |
| anti-human CD209 | 12-2099-42 | PE | 0.012mg/ml | eB-H209 | eBioscience, USA |
| anti-human CD163 | 563697 | FITC | 0.2mg/ml | GHI161 | BD, USA |
| anti-human CD80 | 11-0809-42 | FITC | 0.2mg/ml | 2D10.4 | eBioscience, USA |
| anti-human CD86 | 506957 | PE | 0.05mg/ml | 2331(FUN-1) | BD, USA |
| Table 2. List of mouse flow antibodies | | | | | |

| Antibody | Cat.No | fluorescein | Con | Clone | Vendor |
| --- | --- | --- | --- | --- | --- |
| anti-mouse F4/80 | 123114 | PE-cy7 | 0.2mg/ml | BM8 | Biolegend, USA |
| anti-mouse B7-H4 | 139408 | APC | 0.2mg/ml | HMH4-5G1 | Biolegend, USA |
| anti-mouse B7-H4 | 139405 | PE | 0.2mg/ml | HMH4-5G1 | Biolegend, USA |
| anti-mouse CD206 | 141706 | PE | 0.2mg/ml | C068C2 | Biolegend, USA |
| anti-mouse CD86 | 105007 | PE | 0.2mg/ml | GL-1 | Biolegend, USA |

| anti-mouse IL-10 | 561060 | PE | 0.2mg/ml | JES5-16E3 | BD, USA |  |
| --- | --- | --- | --- | --- | --- | --- |
| anti-mouse iNOS | 17-5920-80 | APC | 0.2mg/ml | CXNFT | eBioscience, USA |  |
| anti-mouse CD80 | 11-8001-82 | FITC | 0.5mg/ml | 16-10A1 | eBioscience, USA |  |
| anti-mouse TNF-α | 12-7321-81 | PE | 0.2mg/ml | MP6-XT22 | eBioscience, USA |  |
| anti-mouse TNF-α | 506304 | FITC | 0.5mg/ml | MP6-XT22 | Biolegend, USA |  |
| anti-mouse Arg-1 | IC5868A | APC | 0.01mg/ml |  | RD, USA |  |
| Table 3. List of Western blotting antibodies | | | | | |  |
| Antibody | Cat.No | Con | Clone | | Vendor | |
| B7-H4 | ab209242 | 0.659mg/ml | EPR20236 | | Abcam, UK | |
| GAPDH | 10494-1-AP | 0.6mg/ml |  | | Proteintech, CHINA | |
| Arg-1 | 16001-1-AP | 0.247mg/ml |  | | Proteintech, CHINA | |
| iNOS | ab202417 | 1.227mg/ml | EP16634 | | Abcam, UK | |
| JAK2 | ab108596 | 1.487mg/ml | EPR108(2) | | Abcam, UK | |
| p-JAK2 STAT1  p-STAT1 | ab32101 10144-2-AP  ab109457 | 0.068mg/ml 0.7mg/ml  0.292mg/ml | E132(Y1007+Y1008)  EPR3147(Y701) | | Abcam, UK Proteintech, CHINA  Proteintech, CHINA | |
| TNF-α | 60291-1-Ig | 1.48mg/ml | 7B8A11 | | Proteintech, CHINA | |
| Antibody | Cat.No | Con | Clone | | Brand | |
| IL-10  PTEN | ab133575  22034-1-AP | 0.307mg/ml | EPR1114 | | Abcam, UK  Proteintech, CHINA | |

# Animals

1. C57BL⁄6 mice (Jinan Pengyue Laboratory Animal Breeding Co. Ltd.) and B7-H4−/− mice (Nanjing Institute of Biomedicine) were bred with sufficient and sterilized food and water, under conditions of controlled temperature (20°C–24°C) and humidity (40%–60%) and a 12 h light/12 h dark cycle in the SPF animal house.
2. The 6–8-week-old females were mated to 8–10-week-old males at a ratio of 2:1. Females with vaginal plugs [gestational day (gd) 0] were segregated every morning.
3. All pregnant mice were randomly divided into uninfected or infected group, with ten mice being included in each group.
4. Ten pregnant B7-H4−/− mice obtained by the same method were set as

the B7-H4-/- infected group.

1. On gd 8, pregnant mice in the infected group and B7-H4-/- infected group were intraperitoneally injected with 400 RH tachyzoites of *T. gondii* in 200

µl sterile phosphate-buffered saline (PBS).

1. The uninfected mice were intraperitoneally injected with 200 µl sterile PBS at the same time.

# Acquisition and Identification of Homozygous B7-H4−/− mice

1. The B7-H4−/− mice were successfully bred by Nanjing University- Nanjing Institute of Biomedicine with the background of C57BL/6.
2. At the age of 4 weeks, the tail of the mice was cut, and the DNA of the tail tissue was obtained using a DNA extraction kit (Generay, 2003G24, China).
3. The DNA was used as a template for real-time PCR amplification.
4. Then, the product was sent to Shanghai Meiji Biomedical Technology Co. Ltd. for DNA sequencing to obtain homozygous mice with B7-H4 gene knockout.
5. The homozygous B7-H4−/− mice were continuously cultivated to guarantee the establishment of an animal model with adverse pregnancy outcomes in infected B7-H4−/− mice.

**Preparation of *T. gondii* (RH Strain)**

1. The RH strain tachyzoites stored in liquid nitrogen were retrieved, and the frozen storage tube was shaken rapidly in a water bath box at 40℃.
2. After complete dissolution, the frozen solution was transferred to the tube with three times the volume of sterile PBS.
3. The mixture was centrifuged at 2800 *× g* for 7 mins at 4 °C.
4. The supernatant was discarded and 400 μl of PBS was added to resuspend the tachyzoites.
5. A trichomonad suspension was stained with 0.5% Evans blue and observed under a microscope, the proportion of hyaline tachyzoites were calculated.
6. The RH strain tachyzoites were cultured in HEp-2 cell lines with Minimum Essential Medium (Bio Channel, BC-M-020, China) with 5% fetal bovine serum (FBS; Gibco, A3160801, United States) and 100 IU/ml penicillin/streptomycin (Solarbio, P1400, China).
7. After 54 h, HEp-2 cells were centrifuged at 400 *× g* for 10 min 4°C, and the clear supernatants were then centrifuged at 2800 *× g* for 7 mins at 4 °C to collect the tachyzoites.
8. The purified tachyzoites were counted in Neubauer chamber and cultured with new HEp-2 cells.

# Scanning Electron Microscopy (SEM)

1. Mice were sacrificed on gd 14 and dissected.
2. All fetuses were carefully removed, washed 5–6 times with 0.1 M phosphate buffer, and then fixed with 2.5% phosphate buffer glutaraldehyde for 48 h at 4°C.
3. The immobilized fetus was dehydrated using a graded ethanol series and soaked for 10 min at a time.
4. The sample was dried by the critical-point technique (Quorum K850, United Kingdom), attached to the sample support, and gold-coated with Quorum Q150RS.
5. All samples were placed on the sample table and observed with a 10 kV scanning electron microscope (ZEISS EVO LS15, Germany).
6. Images were obtained using the SmartSEM user interface software.

# Hematoxylin-Eosin Staining (HE)

1. Pregnant mice were sacrificed on gd 14 and dissected. All fetuses were

carefully removed and the placenta was exposed to 4% paraformaldehyde for one week.

1. Then, it was placed in a specimen box and rinsed with running water for 4-12 h, and dehydrated by a dehydrator.
2. After paraffin embedding, the placenta was cut into 5 µm-thick slices and baked at 55°C-60°C for 3-10 h.
3. Xylene dewaxing of the sample was conducted five times, with each dewaxing lasting for 5–10 min.
4. The placenta was dehydrated by gradient ethanol and soaked three times in steaming water for 3 min.
5. Harris hematoxylin staining was conducted for 10 min, and the slices were then rinsed three times in steaming water for several seconds.
6. 0.5% hydrochloric acid alcohol separation was conducted for 3–10 s, and eosin staining was performed for 2 min.
7. After fixing with xylene, the slides were sealed with neutral resin, covered with cover glass, observed under a microscope and photographed.

# Single Cell Preparation of Mouse

1. Pregnant uninfected, infected and B7-H4-/- infected mice on gd 14 were sacrificed by cervical dislocation.
2. Mouse uteri and placentas were carefully separated and dissected with scissors to remove fetuses, and rinsed twice with sterile cold PBS.
3. The placentas and uteri were then cut into small pieces and shredded carefully by using a Gentle MACS dissociator (Miltenyi, Germany).
4. Tissue suspension was filtered through a 48 µm sterile screen, and the single cell suspension was obtained by gently grinding the needle bolt of the glass syringe as an abrasive rod.
5. The grinding single cell suspension was centrifuged with Ficoll density gradient at 400 *× g* for 20 min, and the white film layer was sucked out,

centrifuged at 400 *× g* for 10 min to wash away the impurities in the white film layer.

1. The mononuclear cells were resuspended with 100-200 μl PBS and then analyzed by flow cytometry.
2. The mouse carcasses were collected in a −20°C refrigerator and disposed by professional organizations.

# Adoptive Transfer Experiment

1. The B7-H4−/− and C57BL⁄6 pregnant mice were sacrificed severally by cervical dislocation on gd 12.
2. Single-cell suspensions were separately prepared from the placental and uterine tissues by cutting the tissues into small pieces and filtering through a 48 µm sterile nylon mesh.
3. The mononuclear cells were isolated by Ficoll density gradient centrifugation.
4. F4/80+ macrophages were positively selected by mouse F4/80 positive selection kit (Thermo Fisher Scientific, 8802-6863, United States) according to the manufacturer’s instructions.
5. The purified macrophages were centrifuged at 400 *× g* for 10 min and then labeled with 15 µM CFSE (MCE, HY-D0938, United States) in Roswell Park Memorial Institute (RPMI) 1640 (Hyclone, SH30809.01, United States) medium without serum in the dark for 15 min under growth conditions.
6. The cells were washed twice in media containing 10% FBS, centrifuged for 10 min at 400 *× g*, resuspended in sterile saline solution, counted, and diluted to 5 × 106 cells per 1 ml.
7. Pregnant B7-H4−/− mice on gd 8 were divided into group1, group2 and group3 randomly, and infected with 200 RH tachyzoites of *T. gondii*.
8. On the same day, the B7-H4−/− mice were injected intravenously with

200 µl sterile saline solution in group1, the B7-H4−/− mice were intravenously treated with 1 × 106 freshly isolated F4/80+ cells from B7- H4−/− and C57BL⁄6 pregnant mice in 200 µl sterile saline solution separately in group2 and group3.

1. Pregnant mice in the three groups were sacrificed by cervical dislocation on gd 14.
2. The pregnancy outcome was observed, monocytes were isolated and analyzed by flow cytometry.

# Collection of Human Clinical Sample

1. Decidual tissues of first trimester (gestational age of 6 to 8 weeks) were obtained from healthy pregnant women who underwent voluntary abortion without any abortifacient or pregnancy complications.
2. The sample collection for this study was approved by the Ethics Committee of Binzhou Medical University, and all subjects were visiting the Department of Obstetrics and Gynecology, Yantai Affiliated Hospital of Binzhou Medical University, Zhifu District Maternal and Child Health Hospital, and Yantai Cancer Hospital.
3. The samples were washed with a sterile saline solution for 5–8 times immediately and saved in Dulbecco’s Modified Eagle’s Medium/high- glucose medium (Hyclone, 12100046, United States) supplemented with 100 IU/mL penicillin/streptomycin.

# Single Cell Preparation of Human

1. The decidual tissues and villi were separated, and the former was cut into pieces and transferred to a broken tube of the single-cell preparation apparatus (Miltenyi, Germany) for crushing.
2. Tissues were digested in 37℃ incubators for 30 min with 0.1% collagenase type IV (Sigma-Aldrich, C4-BIOC, United States) and

25 IU/mL DNase-I (Sigma-Aldrich, 10104159001, United States).

1. The resulting suspension was filtered through 48 µm nylon mesh filters.
2. Mononuclear cells were isolated via density gradient centrifugation using human lymphocyte separation medium (TBD Science, LTS1007, China) at 400 *× g* for 20 min at 20°C in accordance with the manufacturer’s instructions.
3. Approximately 1 × 107 human mononuclear cells were obtained and divided equally into uninfected, infected, and B7-H4-neutralized infected groups.
4. The mononuclear cells were incubated with 10 µg/mL anti-B7-H4 monoclonal antibody (mAb; Thermo Fisher Scientific, 16-5949-82, United States) in B7-H4-neutralized infected group for 1 h.
5. *T. gondii* tachyzoites were added to the infected group and the B7-H4- neutralized infected group at a 2:1 ratio (*T. gondii*: cells).
6. All study samples were cultured in RPMI 1640 medium supplemented with 10% FBS, 100 IU/ml streptomycin, and 100 IU/ml penicillin.
7. After about 22 h culture, mononuclear cells were collected to perform flow analysis.

# Isolation and treatment of Human Decidual Macrophages

1. Mononuclear cells isolated by Ficoll density gradient centrifugation were collected in a flow tube.
2. Decidual macrophages were purified using a human CD14 positive selection kit (Stem Cell Science, #17858, United States) following the manufacturer’s instructions, resulting in purity levels of more than 95%.
3. Approximately 3 × 106 purified human CD14+ decidual macrophages were obtained and divided equally into uninfected, infected, and B7-H4- neutralized infected groups.
4. Approximately 5 × 106 purified human CD14+ decidual macrophages

were obtained and divided equally into uninfected, infected, STAT1- inhibitor infected, B7-H4-neutralized infected, B7-H4-neutralized infected and STAT1-inhibitor groups.

1. The CD14+ decidual macrophages were incubated with 10 µg/mL anti- B7-H4 monoclonal antibody in B7-H4-neutralized infected and B7-H4- neutralized infected and STAT1-inhibitor groups.
2. Cells of the STAT1-inhibitor infected and the B7-H4-neutralized infected and STAT1-inhibitor groups were pre-incubated with Fuldarabine (MCE, HY-B0069, USA).
3. After 2 h, *T. gondii* tachyzoites were added to the infected, B7-H4- neutralized infected, STAT1-inhibitor infected and B7-H4-neutralized infected and STAT1-inhibitor groups at a 2:1 ratio (*T. gondii*: cells).
4. All study samples were cultured in RPMI 1640 medium supplemented with 10% FBS, 100 IU/ml streptomycin, and 100 IU/ml penicillin.
5. After about 22 h of culture, the CD14+ decidual macrophages were collected.

# Phagocytosis Assay

1. Human CD14+ decidual macrophages were suspended at a concentration of 1 × 106 in culture medium, and 300 µl cells of uninfected, infected, and B7-H4-neutralized infected groups were placed into a 24 well plate respectively.
2. The cells were incubated with 10 µg/mL anti-B7-H4 monoclonal antibody in B7-H4-neutralized infected group for 1 h.
3. *T. gondii* tachyzoites were added to the infected and B7-H4-neutralized infected group at a 1:1 ratio (*T. gondii*: cells).
4. After 20 h of culture, the rabbit IgG-FITC complex latex beads (Cayman Chemical Company, 500290, United States) were mixed with macrophages at 37°C for 2 h.
5. Cells were incubated 1 min with trypan blue quenching solution and followed by a wash with assay buffer at 4°C.
6. Subsequently, phagocytic activity of macrophages was photographed by fluorescence microscope and analyzed by flow cytometry.

# Flow Cytometry

**Detection of membrane molecules *in vitro***

1. Human mononuclear cells of the three groups were stained with 7-AAD (KeyGEN NioTECH, KGA219, China) and the following fluorochrome- conjugated mAbs: Pe-cy7-conjugated anti-CD14 and APC-conjugated anti-B7-H4, PE-conjugated anti-CD80, PE-conjugated anti-CD86, FITC- conjugated anti-CD206, FITC-conjugated anti-CD163 at 4°C in the dark for 30 min.
2. The cells were washed and then incubated with 7-AAD (KeyGEN BioTECH, KGA219, China) for 15 mins.
3. Mononuclear cells were washed and resuspended in about 300 μl PBS.
4. Analysis was performed using a FACS canto TM II instrument (BD, United States).

# Detection of Phagocytic ability *in vitro*

1. Purified human decidual macrophages of the three groups were stained with PE-cy7-conjugated anti-CD14 at 4°C in the dark for 30 min.
2. The cells were washed and then incubated with 7-AAD for 15 mins.
3. Mononuclear cells were washed and resuspended in about 300 μl PBS.
4. Analysis was performed using a FACS canto TM II instrument.

# Detection of membrane molecules *in vivo*

1. The mouse mononuclear cells were stained with the following mouse- specific mAbs: PE-cy7-conjugated anti-F4/80, APC-conjugated anti-B7-

H4, PE-conjugated anti-CD86, PE-conjugated anti-CD206, FITC- conjugated anti-CD80, PE-conjugated anti-CD80 at 4°C in the dark for 30 min and then washed once.

1. The mononuclear cells were resuspended in about 300 μl PBS and analyzed by flow cytometry.

**Detection of enzymes *in vivo***

1. The mouse mononuclear cells were stained with the following mouse- specific mAbs: PE-cy7-conjugated anti-F4/80, PE-conjugated anti-B7-H4, at 4°C in the dark for 30 min and then washed once.
2. We fixed and permeabilized the cells in 1× Fix/Perm buffer (Thermo Fisher Scientific, 00-5523-00, United States) for 30 min at 4°C in accordance with the protocol and washed twice.
3. The cells were incubated with mouse-specific mAbs: APC-conjugated anti-Arg-1 and APC-conjugated anti-inducible nitric oxide synthase (iNOS) at 4°C in the dark for 40 min and washed once.
4. The mononuclear cells were resuspended in about 300 μl PBS and analyzed by flow cytometry.

**Detection of cytokines *in vivo***

1. The mononuclear cells were cultured for 4–6 h in a leukocyte activation cocktail (eBioscience, 51-20421E, USA) before adding mAbs of cytokines.
2. Cells were stained with the following mouse-specific mAbs: PE-cy7- conjugated anti-F4/80, APC-conjugated anti-B7-H4, at 4°C in the dark for 30 min and then washed once.
3. We fixed and permeabilized the cells in 1× Fix/Perm buffer for 30 min at 4°C in accordance with the protocol and washed twice.
4. The cells were incubated with mouse-specific mAbs: PE-conjugated anti-IL-10, FITC-conjugated anti-TNF-α at 4°C in the dark for 40 min and

washed once.

1. Analysis was performed using a FACS canto TM II instrument.

# Western Blotting

1. The CD14+ decidual macrophages from each group were incubated for 24 h and lysed using ice-cold radioimmunoprecipitation lysis buffer (RIPA; Beyotime, P0013B, China) and phenylmethanesulfonyl fluoride (PMSF; Beyotime, ST506-2, China).
2. The cells were split on ice for 40 min and then centrifuged for 20 min at 12, 000 *× g* to remove the debris at 4°C.
3. The concentration of protein extracts was determined using a bicinchoninic acid protein assay kit (Solarbio, PC00020, China) and boiled in 5× SDS-PAGE (Beyotime, P0015, China) sample loading buffer for 8 min.
4. The total protein (30 µg) was separated by 12% sodium dodecyl sulfate- polyacrylamide gel electrophoresis (Beyotime, P0012AC, China) and transferred to polyvinylidene difluoride (PVDF) membranes (Merck Millipore, ISEQ00010, Germany).
5. The membranes were blocked for 2.5 h in 5% non-fat dry milk in Tris- buffered saline with Tween-20 at room temperature (20°C–25°C).
6. Then, the PVDF membranes were incubated overnight with the primary antibodies for B7-H4 (1:2000), CD80 (1:1000), CD86 (1:1000, Proteintech), CD163 (1:2000), CD206 (1:1000), Arg-1 (1:1000), iNOS (1:1000), JAK2 (1:1000), p-JAK2 (1:1000), STAT1 (1:2000), and p- STAT1 (1:2,000), TNF-α (1:2000), IL-10 (1:1000); glyceraldehyde-3- phosphate dehydrogenase (GAPDH) (1:40, 000) at 4°C.
7. The membranes were incubated with appropriate secondary antibodies for 2 h at room temperature, and electroluminescence was detected using an enhanced chemiluminescence kit (Wanleibio, WLA006c, China).
8. Protein expression levels were determined using ImageJ. GAPDH was used as the internal control.

# Immunofluorescence

1. Purified human CD14+ decidual macrophages from uninfected, infected and B7-H4-neutralized infected groups were air-dried onto Polysine microscope adhesion slides.
2. After fixation in 4% paraformaldehyde for 30 min, the slides were then blocked with goat serum for 1 h at room temperature.
3. The cells were incubated overnight at 4°C with anti-B7-H4 (1:200), anti- IL-10 (1:200) and anti-TNF-α (1:200).
4. After washing three times with PBS, the cells were incubated with appropriate concentrations of secondary antibodies for 1 h at 37°C.
5. Subsequently, the cells were stained with the nucleic acid stain 4’, 6- diamidino-2-phenylindole for 15 min and washed.
6. A drop of anti-fluorescence quenching agent were added to seal the tablet.
7. Finally, confocal microscopy of cells was performed using Zeiss LSM880.
