# Supplementary figures and images for: Effect of B7-H4 downregulation induced by Toxoplasma gondii infection on dysfunction of decidual macrophages contributes to adverse pregnancy outcomes
Source: Parasit Vectors. 2022 Dec 13;15:464. doi: 10.1186/s13071-022-05560-9 (PMC9746109; doi:10.1186/s13071-022-05560-9)

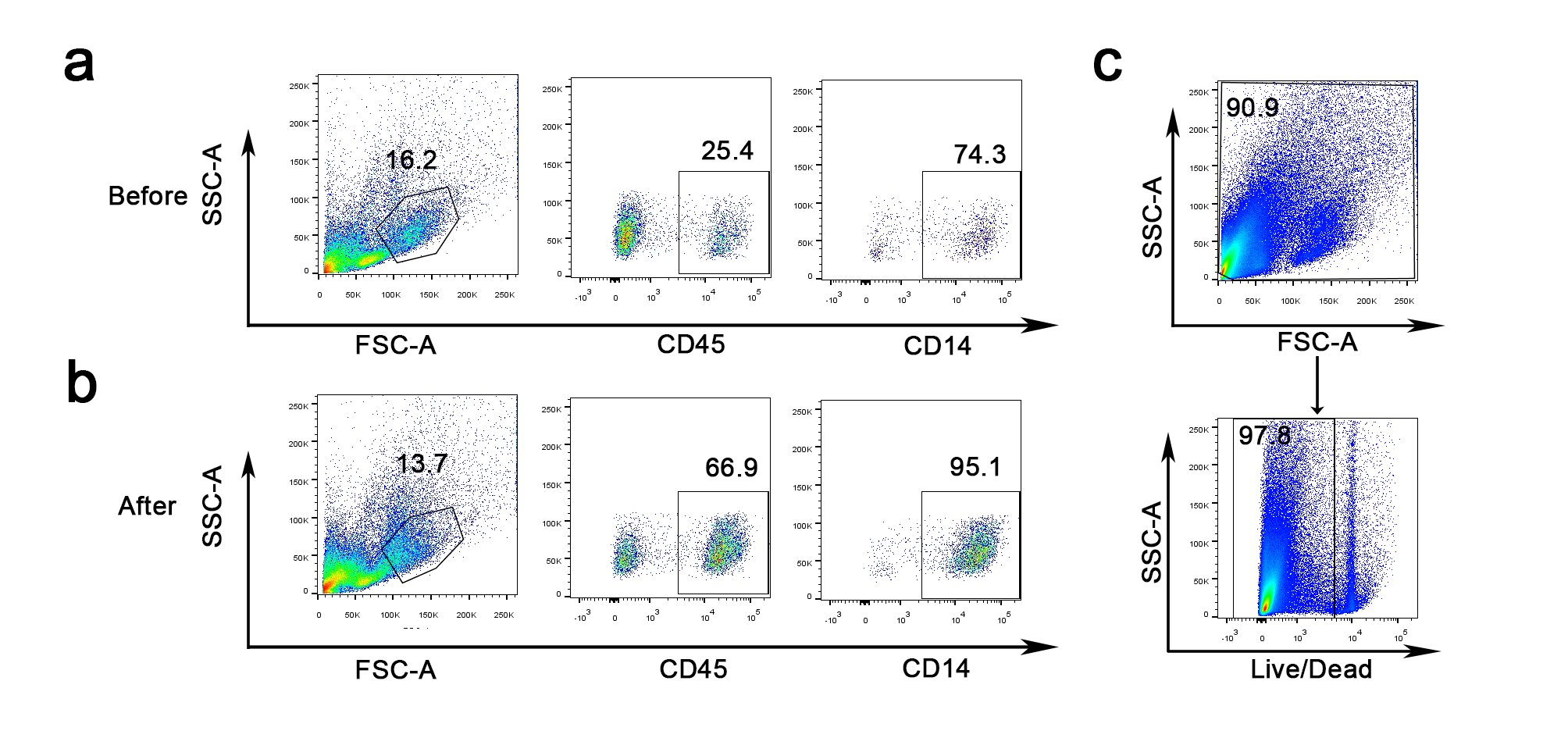

Supplement: Supplementary file 2 — Additional file 2: Figure S1. The purity of human decidual macrophages. a Flow cytometry results showing the percentage of human decidual macrophages before purified by human CD14 positive selection kit. b Flow cytometry results showing the percentage of human decidual macrophages after purified by human CD14 positive selection kit. c The proportion of live cells in mouse. [file 13071_2022_5560_MOESM2_ESM.tif]

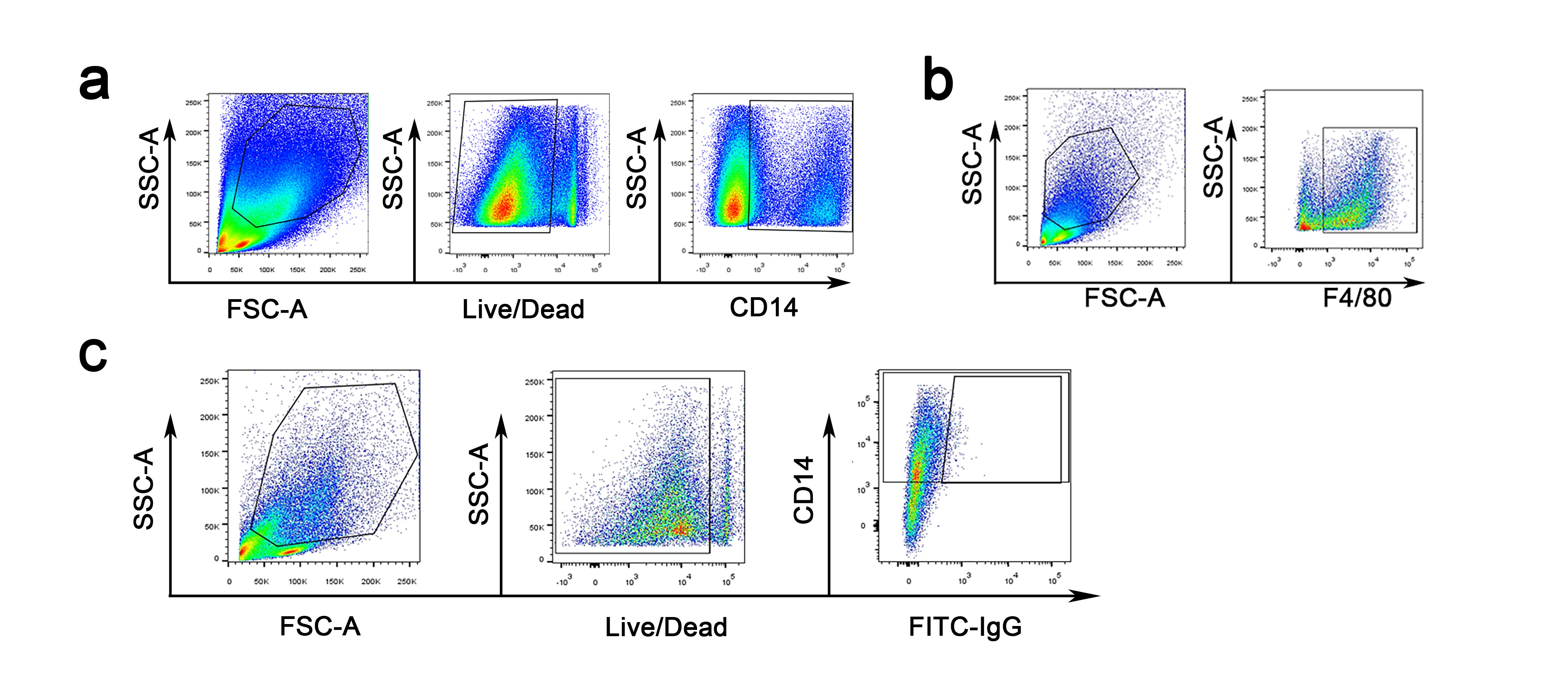

Supplement: Supplementary file 3 — Additional file 3: Figure S2. a Gating strategy for flow cytometry in the human experiment. b Gating strategy for flow cytometry in mice experiment. c Gating strategy for flow cytometry in phagocytosis assay experiment of human decidual macrophages. [file 13071_2022_5560_MOESM3_ESM.tiff]
